# Optogenetics in neurobiology and neurodegenerative disorders: from neural circuit mechanisms to precision neuromodulation

**DOI:** 10.3389/fmedt.2026.1958917

**Published:** 2026-10-29

**Authors:** V. Singaravel

**Affiliations:** Department of Biochemistry, Saveetha Institute of Basic Medical Sciences (SIBMS), Saveetha Institute of Medical and Technical Sciences (SIMATS), Chennai, Tamil Nadu, India

**Keywords:** microbial opsins, neural circuits, neurodegenerative disorders, optogenetics, precision neuromodulation, synaptic plasticity

## Abstract

Neurodegenerative disorders, including Alzheimer's disease, Parkinson's disease, Huntington's disease, amyotrophic lateral sclerosis, multiple sclerosis and stroke, are characterized by progressive neuronal dysfunction, synaptic impairment and neural circuit degeneration, leading to severe cognitive and motor deficits. Although considerable progress has been made in understanding their molecular pathology, the circuit level mechanisms driving disease onset and progression remain incompletely understood. Optogenetics, which combines microbial opsins, genetic engineering and optical stimulation, enables reversible, cell-specific control of neuronal activity with millisecond precision and has transformed neuroscience from observational analysis to causal investigation of neural circuits. This review summarizes recent advances in the molecular basis of optogenetics, microbial opsins, gene delivery strategies and optical stimulation technologies, together with their applications in basic neurobiology and neurodegenerative disease research. We discuss how optogenetics has advanced understanding of neural circuit organization, synaptic plasticity, memory, neurodevelopment, sleep regulation, protein aggregation, synaptic dysfunction, neuroinflammation, mitochondrial impairment, oxidative stress and regulated cell death. Although these studies have generated important mechanistic insights and demonstrated functional improvements in experimental models, translation into human therapies remains investigational due to challenges associated with gene delivery, long-term opsin expression, immune compatibility and optical accessibility. Emerging therapeutic strategies, including closed-loop neuromodulation, wireless photostimulation, artificial intelligence-assisted neural decoding, nanotechnology enabled light delivery and brain computer interfaces, are critically evaluated with emphasis on their current evidence status, translational challenges and limitations for clinical implementation. Collectively, optogenetics provides an unprecedented platform for deciphering disease mechanisms and evaluating circuit-based neuromodulation strategies, while continued technological and biological validation will determine its future role in precision therapies for neurodegenerative disorders.

## 1 Introduction

Neurodegenerative disorders represent one of the most formidable challenges to modern medicine owing to their progressive nature, increasing prevalence, and profound socioeconomic impact (1, 2). Characterized by the gradual loss of neuronal structure and function, these disorders encompass a diverse group of diseases, including Alzheimer's disease, Parkinson's disease, amyotrophic lateral sclerosis, Huntington's disease and multiple sclerosis (2–5). Collectively, these conditions affect hundreds of millions of individuals worldwide and constitute a major cause of disability, cognitive decline, impaired quality of life, and premature mortality (1, 6). The rapid expansion of the aging population has further intensified the global burden of neurodegeneration, placing unprecedented pressure on healthcare systems and highlighting the urgent need for innovative approaches that not only alleviate symptoms but also elucidate disease mechanisms and enable effective therapeutic intervention (2, 7).

Despite remarkable advances in molecular neuroscience, the pathogenesis of neurodegenerative diseases remains incompletely understood (8, 9). Although each disorder exhibits distinct pathological hallmarks, they share several convergent mechanisms, including aberrant protein aggregation, mitochondrial dysfunction, oxidative stress, excitotoxicity, impaired synaptic transmission, chronic neuroinflammation, disrupted axonal transport, and progressive neuronal death (3, 10). These interconnected processes ultimately compromise the integrity of neuronal circuits that govern cognition, movement, memory, emotion, and sensory processing (11, 12). Consequently, considerable research efforts have shifted from investigating isolated molecular pathways to understanding how dysfunction within specific neural circuits contributes to disease initiation, progression, and clinical manifestation (10, 13).

Traditional experimental approaches have provided invaluable insights into nervous system function; however, each possesses inherent limitations (14, 15). Pharmacological interventions frequently lack cellular specificity and often produce widespread off-target effects owing to the diffuse distribution of neurotransmitter receptors (16). Electrical stimulation techniques, including deep-brain stimulation (DBS), have demonstrated clinical efficacy in selected neurological disorders, particularly movement disorders, yet they indiscriminately activate multiple neuronal populations and passing axonal fibers, thereby limiting mechanistic interpretation and therapeutic precision (17, 18). Likewise, conventional lesion studies and electrophysiological recordings are often unable to establish direct causal relationships between the activity of genetically defined neuronal populations and complex behavioural or pathological outcomes (10, 16). These limitations underscore the need for technologies capable of manipulating neural activity with exceptional temporal, spatial, and cellular specificity (16, 19).

Optogenetics has emerged as one of the most transformative innovations in modern neuroscience by enabling precise optical control of genetically defined cells with millisecond temporal resolution (13, 19). The technology integrates molecular genetics, microbial photobiology, optical engineering, viral gene delivery, and systems neuroscience to achieve reversible activation or inhibition of specific neuronal populations using light-sensitive proteins known as microbial opsins (15, 16). Following targeted expression of opsins such as channelrhodopsins, halorhodopsins, archaerhodopsins, and their engineered derivatives, neuronal activity can be manipulated through illumination at defined wavelengths, allowing investigators to interrogate neural circuit function with unprecedented precision (14, 20). Unlike conventional neuromodulation approaches, optogenetics permits selective excitation or silencing of individual neuronal subtypes, projection-specific pathways, or even subcellular compartments while preserving the surrounding neural architecture (13, 15, 16). This unique combination of genetic specificity and optical precision has fundamentally transformed experimental neuroscience by enabling causal analyses of brain function rather than simple observational correlations (10, 16). The historical evolution of optogenetics, from the discovery of microbial opsins and the development of light-gated ion channels to advanced neuromodulation platforms and emerging clinical applications, is summarized in Figure 1.

**Figure 1:**
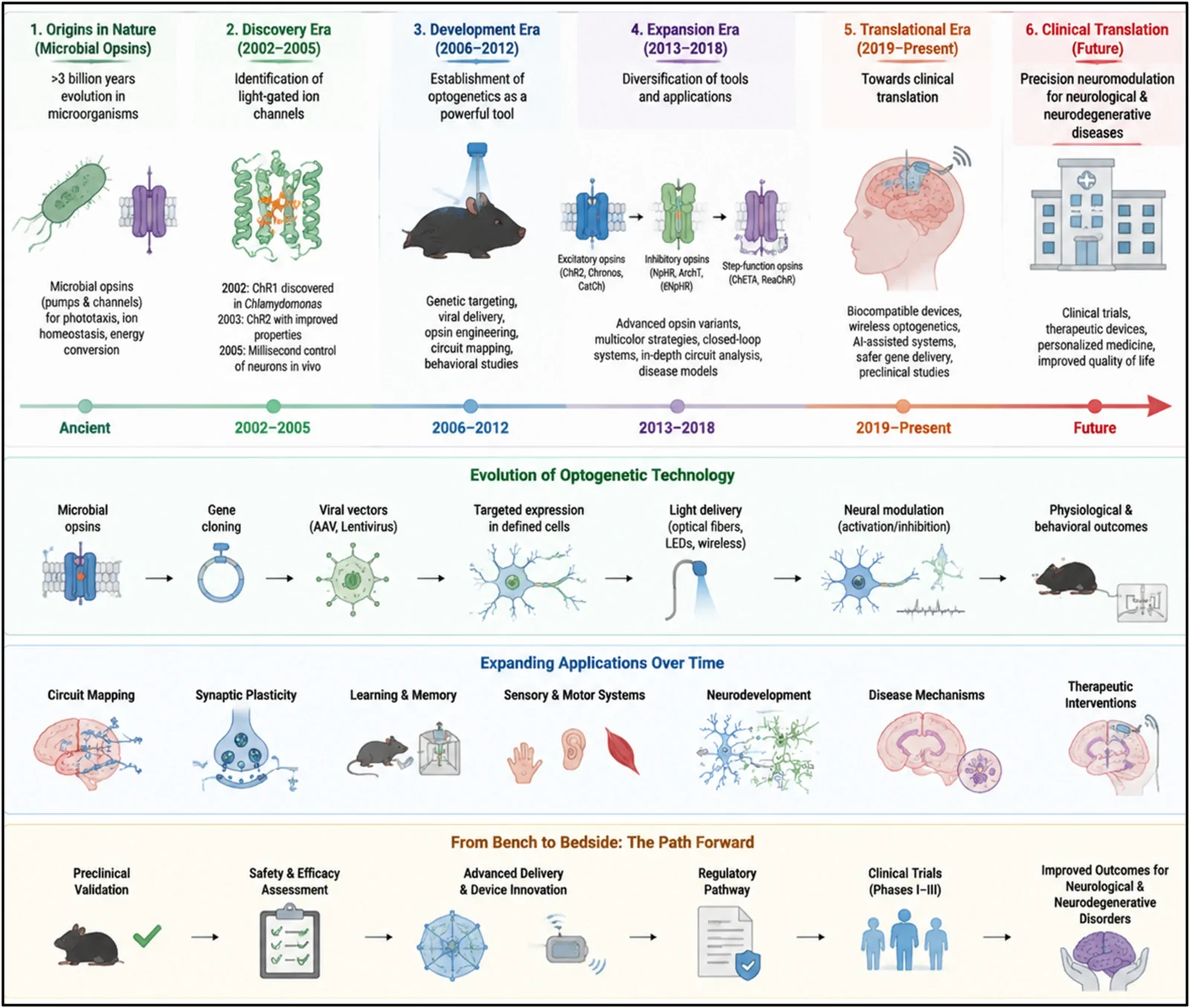
Evolution of optogenetics from microbial opsins to clinical translation. Schematic illustration depicting the historical progression of optogenetics from the discovery of microbial rhodopsins and channelrhodopsins to the development of advanced opsin engineering, viral gene delivery systems, optical stimulation technologies, neural circuit interrogation, and emerging clinical applications in precision neuromodulation for neurological and neurodegenerative disorders.

Since the pioneering demonstration of light-activated neuronal control in mammalian neurons, optogenetics has evolved rapidly through advances in protein engineering, viral vector technology, optical instrumentation, and computational neuroscience (16, 19). Successive generations of engineered opsins now exhibit enhanced light sensitivity, faster kinetics, improved ion conductance, greater spectral diversity, and superior tissue penetration through red-shifted activation wavelengths (21, 22). Simultaneously, innovations in adeno-associated viral vectors, cell type-specific promoters, wireless light-delivery systems, micro-light-emitting diode (micro-LED) devices, gradient-index lenses, holographic stimulation, and two-photon excitation have substantially expanded the experimental versatility of optogenetics (23, 24). Furthermore, integration with complementary technologies including calcium imaging, genetically encoded voltage indicators, single-cell transcriptomics, spatial omics, functional magnetic resonance imaging, artificial intelligence-assisted neural decoding, and closed-loop neuromodulation has established optogenetics as a central platform for investigating brain function across multiple spatial and temporal scales (23, 25).

Beyond its foundational role in basic neuroscience, optogenetics has revolutionized the investigation of neural circuits underlying behaviour, cognition, learning, memory, emotion, sensory perception, motor coordination, sleep regulation, and social interaction (10, 16). By providing direct causal evidence linking neuronal activity to behavioural outcomes, optogenetics has facilitated the identification of previously inaccessible circuit-level mechanisms responsible for normal brain function and neurological disease (14, 23). More importantly, these advances have reshaped contemporary concepts of neurodegeneration by demonstrating that pathological dysfunction frequently arises from alterations in dynamic neuronal networks rather than isolated cellular abnormalities alone (25, 26).

In the context of neurodegenerative disorders, optogenetics has become an indispensable experimental platform for dissecting disease-specific neural circuits and evaluating novel therapeutic strategies (10, 25). Experimental studies have demonstrated that selective modulation of hippocampal neuronal ensembles can restore memory performance and enhance gamma oscillatory activity in models of Alzheimer's disease (26, 27), while precise regulation of basal ganglia circuitry has improved motor dysfunction in Parkinson's disease (28). Similar approaches have revealed mechanistic insights into corticospinal degeneration in amyotrophic lateral sclerosis, corticostriatal dysfunction in Huntington's disease, cortical reorganization following ischemic stroke, and remyelination-associated neural plasticity in multiple sclerosis (10, 25). Collectively, these investigations have significantly advanced understanding of disease pathophysiology while identifying promising circuit-based therapeutic targets that may complement or surpass existing pharmacological interventions (16, 25).

Despite these remarkable achievements, several obstacles continue to impede the clinical translation of optogenetics (23, 25). Safe and efficient gene delivery, long-term opsin expression, immune responses to viral vectors, limited penetration of visible light into deep-brain tissues, device biocompatibility and regulatory considerations remain major challenges (7, 25). Research on red-shifted and near-infrared-responsive opsins, wireless photonic implants, nanotechnology-assisted light delivery, gene-editing platforms and artificial intelligence-driven adaptive neuromodulation is investigating potential ways to address these limitations (21, 25). Although these advances expand experimental capacities, their effectiveness and safety as therapeutic approaches for precision neurology and neurorehabilitation remain to be established (23, 25).

Although numerous reviews have comprehensively discussed specific aspects of optogenetics, including microbial opsin engineering, neural circuit interrogation, optical stimulation technologies, retinal applications, and disease-specific studies, these contributions have generally focused on individual technological or pathological themes. Previous reviews have provided important foundations for understanding optogenetic principles, emerging optical platforms and neurological applications (23, 25); however, fewer studies have systematically integrated molecular engineering advances, circuit-level mechanisms, disease-specific evidence, and translational challenges within a single framework. As summarized in Table 1, the present review differs from previous studies by critically evaluating not only the capabilities of optogenetic technologies but also the maturity of experimental evidence, limitations of current disease models, and barriers that influence clinical translation. Therefore, this review aims to provide an evidence-based perspective on the progression of optogenetics from a powerful experimental neuroscience tool toward a potential precision neuromodulation strategy.

**Table 1:** Comparison of the present review with previous optogenetics reviews and its unique scope.

Review focus	Previous reviews mainly covered	Limitation addressed in present review	References
Opsin engineering	Molecular structure, spectral properties, kinetics	Links engineering advances with translational requirements	(15, 16, 21)
Neural circuits	Circuit mapping and behavioural neuroscience	Evaluates disease relevance and therapeutic interpretation	(10, 23)
Neurodegenerative disorders	Individual disease applications	Compares evidence maturity across diseases	(10, 26, 28, 50)
Optical technologies	Devices, wireless systems, light delivery	Discusses realistic clinical constraints	(23, 109, 117)
AI, nanotechnology, BCI	Emerging concepts	Separates demonstrated evidence from future possibilities	(101, 103, 106, 108, 109)
Clinical translation	General therapeutic potential	Evaluates regulatory, biological, and engineering barriers	(48, 100, 113, 117)

Therefore, this review provides a comprehensive and critical assessment of contemporary advances in optogenetics and its applications in neurobiology and neurodegenerative disorders, with particular emphasis on integrating molecular engineering, neural circuit mechanisms, technological innovations and translational considerations. The review first discusses the molecular principles, genetic engineering strategies and optical technologies underpinning optogenetic neuromodulation. It then examines the role of optogenetics in deciphering neural circuit organization and fundamental neurobiological processes before critically evaluating its applications across major neurodegenerative diseases, including Alzheimer's disease, Parkinson's disease, Huntington's disease, amyotrophic lateral sclerosis, multiple sclerosis and stroke. Finally, emerging optogenetic platforms, including closed-loop systems, wireless technologies, artificial intelligence-assisted approaches, nanotechnology-enabled delivery strategies, and regenerative approaches, are discussed with emphasis on current evidence status, translational challenges, ethical considerations and future research directions. This review aims to distinguish established experimental applications from potential therapeutic opportunities and provide a balanced perspective on the future development of optogenetics as a precision neuromodulation strategy.

## 2 Historical development of optogenetics

### 2.1 Discovery of microbial opsins

The conceptual foundation of optogenetics emerged from interdisciplinary research in microbiology, photobiology, biophysics and neuroscience. Microbial opsins are retinal-binding transmembrane proteins that convert light into electrical signals by functioning as light-gated ion channels or pumps, unlike vertebrate visual opsins that require G protein-coupled signaling. Their ability to directly regulate membrane potential established the molecular basis of modern optogenetics (16, 29).

The first major breakthrough occurred in 1971 when Dieter Oesterhelt and Walther Stoeckenius discovered bacteriorhodopsin in *Halobacterium salinarum*. Acting as a light-driven proton pump, bacteriorhodopsin converts solar energy into proton gradients for ATP synthesis and became the first microbial rhodopsin shown to possess direct photoelectrical activity (29). Structural studies subsequently revealed a conserved architecture of seven transmembrane *α*-helices surrounding a retinal chromophore linked through a Schiff base. Light-induced retinal isomerization initiates conformational changes that drive ion transport, establishing the structural and functional principles common to microbial opsins (30). Subsequent discoveries identified halorhodopsin, a light-driven chloride pump and sensory rhodopsins, which mediate phototaxis through intracellular signaling pathways (31). Together, these proteins demonstrated remarkable functional diversity, including proton pumping, chloride transport and sensory signaling, while exhibiting rapid activation, photostability and reversible switching properties that later proved ideal for neuromodulation. For several decades, microbial opsins were studied primarily within microbial physiology. Nevertheless, advances in crystallography, spectroscopy, electrophysiology and molecular genetics clarified their retinal photochemistry, ion selectivity, photocycle kinetics, and conformational dynamics, providing a framework for engineering proteins with altered spectral sensitivity, conductance and kinetics (30, 32).

A major breakthrough occurred in 2002 with the discovery of Channelrhodopsin-1 (ChR1) in Chlamydomonas reinhardtii, identified as a directly light-gated cation channel mediating algal phototaxis (33). Unlike bacteriorhodopsin, ChR1 functions as a passive ion channel, allowing rapid influx of monovalent and divalent cations following blue-light stimulation. One year later, Channelrhodopsin-2 (ChR2) was identified with faster kinetics, larger photocurrents, and superior conductance, making it highly suitable for controlling neuronal excitability (34). Although initially regarded as proteins of interest in algal biology, neuroscientists quickly recognized that ChR2 could provide genetically targeted optical control of neuronal firing with millisecond precision. This discovery bridged microbial photobiology and neuroscience, overcoming the limitations of conventional electrical and pharmacological stimulation and laying the foundation for optogenetics (16, 19). Today, microbial opsins have evolved from biological curiosities into indispensable tools for neuroscience. Extensive genomic discovery and protein engineering have generated hundreds of natural and synthetic opsins with diverse spectral properties, ion selectivity, kinetics, and subcellular targeting, forming the molecular basis of next-generation precision neuromodulation.

## 3 Fundamentals of optogenetics

### 3.1 Principles of optogenetic neuromodulation

Optogenetics enables reversible, cell type-specific control of neuronal activity through the integration of molecular genetics and optical stimulation. Unlike pharmacological or electrical neuromodulation, which often lacks temporal and cellular specificity, optogenetics manipulates genetically defined neurons with millisecond precision and micrometre-scale spatial resolution. By combining microbial opsins with targeted gene delivery and controlled light stimulation, it enables causal investigation of neural circuits underlying behaviour, cognition, sensory processing, motor function, and neurological disease (15, 16). The technology relies on expression of microbial opsins within selected neuronal populations. Upon illumination, retinal-bound opsins undergo rapid conformational changes that regulate ion flux across the plasma membrane, producing either neuronal depolarization or hyperpolarization depending on the opsin employed. This bidirectional modulation permits precise investigation of functional connectivity and establishes direct causal relationships between neuronal activity and physiological or behavioural outcomes (14, 19). Effective optogenetic neuromodulation requires four integrated components: genetically encoded opsins, targeted gene delivery, optical stimulation, and functional assessment. Opsin genes are introduced using viral vectors, transgenic models, or genome-editing approaches under cell-specific promoters. Following expression, light activation triggers rapid ion transport, altering membrane potential, neurotransmitter release, and network activity. Functional responses are evaluated using electrophysiology, calcium imaging, voltage indicators, behavioural analysis, and multimodal neuroimaging (15, 23).

A major advantage of optogenetics is its exceptional temporal fidelity. Most microbial opsins activate and deactivate within milliseconds, allowing precise control of action potentials, burst firing, oscillatory synchronization, and synaptic plasticity. In contrast, pharmacological interventions exhibit slower kinetics, while electrical stimulation lacks cellular specificity by activating both neuronal somata and passing axons. Optogenetics overcomes these limitations through genetically restricted expression using cell-specific promoters, Cre-lox recombination, and intersectional genetic strategies, enabling selective manipulation of neuronal subtypes defined by neurotransmitter phenotype, developmental lineage, or projection targets (15, 35). Beyond regulating neuronal firing, optogenetics has transformed investigation of synaptic connectivity, long-term potentiation, long-term depression, spike timing-dependent plasticity, and neural network dynamics. It has shifted neuroscience from correlative observations to direct causal analysis of neuronal circuits responsible for memory, sensory integration, emotional regulation, and motor behaviour (10, 16). Continuous engineering of microbial opsins has expanded the optogenetic toolbox to include excitatory and inhibitory channels, proton pumps, bistable variants, and red-shifted proteins with improved tissue penetration. Combined with calcium imaging, voltage sensors, single-cell and spatial transcriptomics, artificial intelligence, and closed-loop neuromodulation, optogenetics has become a comprehensive platform for investigating brain function across molecular, cellular, circuit, and behavioural levels (23, 25). Its applications now extend beyond basic neuroscience to models of Alzheimer's disease, Parkinson's disease, epilepsy, Huntington's disease, amyotrophic lateral sclerosis, stroke, chronic pain, depression, and anxiety disorders. Although clinical translation remains challenged by gene delivery, light penetration and device biocompatibility, advances in red-shifted opsins, wireless photonic systems and nanotechnology provide experimental avenues for exploring precision neuromodulation, although therapeutics effectiveness remains unconfirmed (24, 25).

### 3.2 Microbial opsins and molecular mechanisms

Microbial opsins constitute the molecular basis of optogenetics and represent one of the best-characterized families of photoreceptor proteins. These retinal-binding membrane proteins are widely distributed in archaea, bacteria, fungi, and unicellular algae, where they regulate phototaxis, ion homeostasis, osmotic balance, and energy conversion (32, 36). Unlike vertebrate visual opsins, which activate G protein-coupled signaling cascades, microbial opsins directly convert light into ion transport across the plasma membrane, enabling rapid electrical responses ideally suited for optical control of neuronal activity. Structurally, microbial opsins share a conserved architecture comprising seven transmembrane *α*-helices surrounding a retinal chromophore linked to a conserved lysine residue through a protonated Schiff base. Photon absorption induces retinal photoisomerization from the all-trans to the 13-cis configuration, initiating a photocycle that alters ion channel or pump conformation and produces membrane depolarization or hyperpolarization depending on the opsin type. This process occurs within milliseconds, providing exceptional temporal precision, photostability, and repetitive activation with minimal functional decline (30, 32).

Functional diversity arises from variations within the retinal-binding pocket and ion-conducting pathway that determine ion selectivity, spectral sensitivity, conductance, activation kinetics, and recovery rates. Consequently, microbial opsins include proton pumps, chloride pumps, cation channels, anion channels, and sensory photoreceptors, each possessing unique electrophysiological properties. Recent advances in cryo-electron microscopy, structural biology, molecular dynamics simulations, and protein engineering have further clarified opsin conformational dynamics, enabling rational design of next-generation optogenetic tools with enhanced performance and therapeutic potential (36, 37).

#### 3.2.1 Channelrhodopsins

Channelrhodopsins are the principal excitatory optogenetic actuators and remain the cornerstone of modern optogenetics. Initially identified in the green alga Chlamydomonas reinhardtii, these light-gated cation channels mediate phototaxis by converting blue-light stimulation into rapid membrane depolarization (33, 34). Unlike bacteriorhodopsins and halorhodopsins, which actively pump ions across membranes, channelrhodopsins function as passive ion channels that transiently permit diffusion of sodium, potassium, calcium, and hydrogen ions according to their electrochemical gradients. The discovery of Channelrhodopsin-1 (ChR1) in 2002 demonstrated the feasibility of optical membrane depolarization; however, its relatively small photocurrents and limited expression restricted its application in neuroscience (33). Identification of Channelrhodopsin-2 (ChR2) in 2003 marked a major breakthrough because of its faster activation kinetics, greater conductance, and improved membrane trafficking, enabling reliable optical stimulation of mammalian neurons (34). In 2005, Boyden and colleagues demonstrated millisecond optical control of neuronal firing using ChR2, establishing the fundamental principles of optogenetics and initiating a new era of circuit neuroscience (19).

At the molecular level, ChR2 is composed of seven transmembrane helices surrounding a central ion-conducting pore. Blue-light illumination (∼470 nm) induces retinal photoisomerization, producing conformational changes that transiently open the channel and permit influx of Na⁺, K⁺, Ca²⁺, and H⁺ ions. The resulting inward current depolarizes neurons, triggers action potentials, and enables precise optical control of neuronal firing with millisecond resolution (30, 37). Extensive protein engineering has generated numerous ChR2 variants with improved properties. ChETA exhibits accelerated channel closure kinetics, supporting stimulation frequencies exceeding 200 Hz and enabling studies of fast-spiking interneurons (38). CatCh displays enhanced calcium permeability and larger photocurrents, reducing light intensity requirements while improving neuronal activation (39). Comparative genomic studies have identified additional channelrhodopsins, including Chronos, CoChR, and PsChR, which possess distinct spectral sensitivities, conductance profiles, and kinetic characteristics. Among these, Chronos combines exceptionally rapid activation with high light sensitivity, permitting efficient neuronal stimulation while minimizing tissue heating (21). Channelrhodopsins have transformed systems neuroscience by enabling selective activation of genetically defined neuronal populations, precise mapping of synaptic connectivity, characterization of neurotransmitter release, and investigation of neuronal oscillations underlying cognition, memory, emotion, and behaviour. Projection-specific stimulation further permits interrogation of long-range axonal pathways without activating neighbouring neuronal somata, providing unprecedented insight into distributed neural networks (10, 16).

Beyond basic neuroscience, channelrhodopsins have demonstrated considerable translational potential. Experimental studies have employed ChR2-mediated stimulation to restore hippocampal memory circuits in Alzheimer's disease, modulate basal ganglia function in Parkinson's disease, suppress seizures through activation of inhibitory interneurons, and promote functional recovery after spinal cord injury and ischemic stroke. Consequently, channelrhodopsins continue to serve as both indispensable experimental tools and promising therapeutic actuators for precision neuromodulation.

## 4 Optogenetics in neurobiology

Optogenetics has revolutionized neurobiology by enabling precise, reversible, and cell type specific manipulation of genetically defined neuronal populations with exceptional temporal and spatial resolution. This technology has transformed investigations of neural circuit organization, synaptic plasticity, learning and memory, sensorimotor integration, neurodevelopment, and sleep regulation, providing direct causal insights into brain function and neural network dynamics. The major neurobiological applications of optogenetics discussed in this section are illustrated in Figure 2, highlighting its broad impact on understanding both physiological and pathological neural processes.

**Figure 2:**
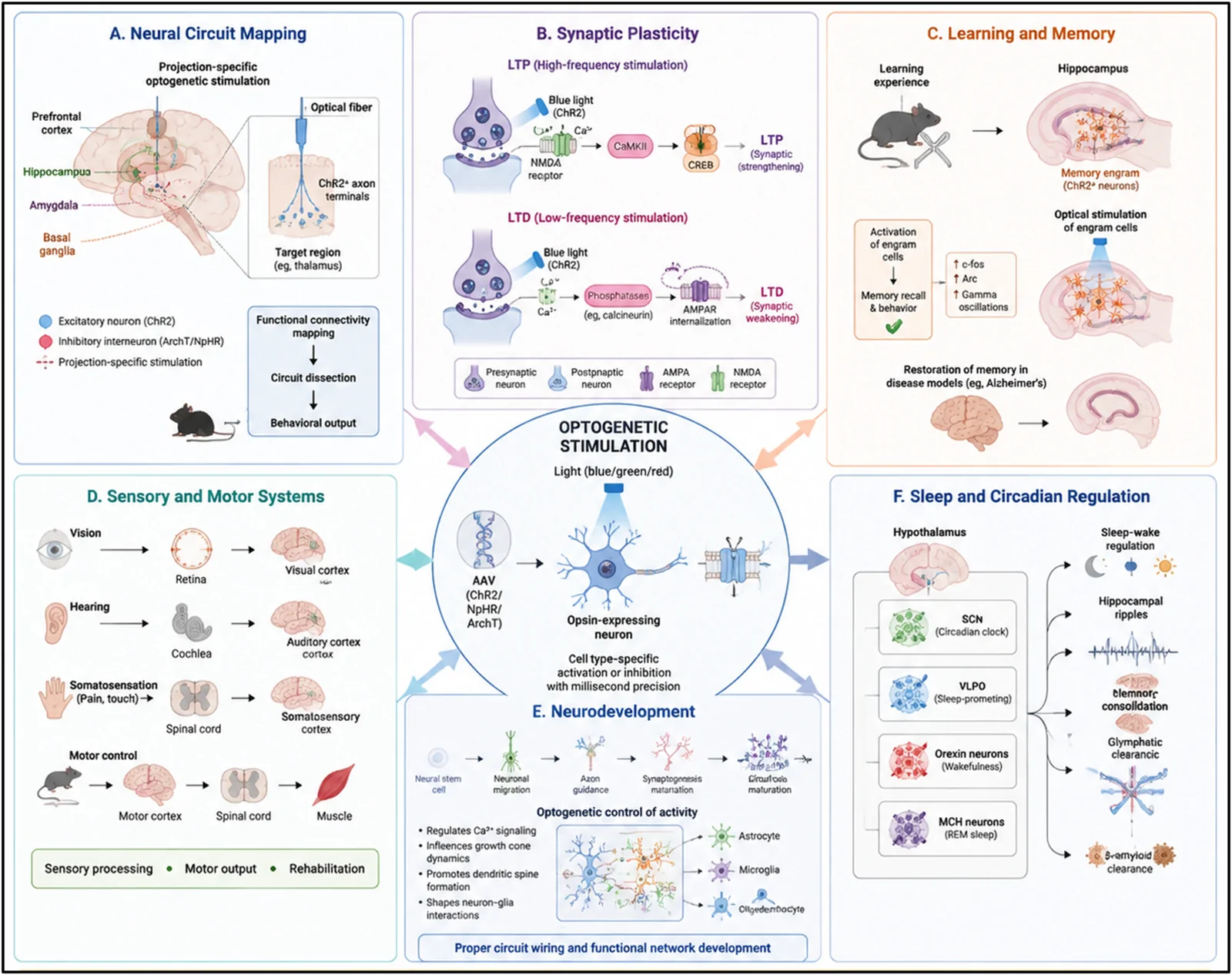
Applications of optogenetics in basic neurobiology. Optogenetics enables precise, cell type specific manipulation of genetically defined neuronal populations to investigate fundamental neurobiological processes. Projection specific stimulation facilitates neural circuit mapping and functional connectivity analysis **(A)**, while selective activation of synaptic pathways elucidates the mechanisms underlying long-term potentiation and long-term depression **(B)** Optical manipulation of hippocampal engram cells reveals the neural basis of learning and memory **(C)** Optogenetics further advances studies of sensory processing, motor control, and rehabilitation **(D)**, neurodevelopment and circuit maturation **(E)**, and sleep, circadian regulation, memory consolidation, and glymphatic clearance **(F)**, providing a powerful platform for understanding brain function and neurological disorders.

### 4.1 Neural circuit mapping

One of the most transformative contributions of optogenetics to modern neuroscience has been its ability to establish direct causal relationships between neuronal activity and circuit function. Before the advent of optogenetics, investigations of neural circuitry relied primarily on electrophysiological recordings, pharmacological manipulations, lesion studies, and anatomical tracing techniques. Although these methodologies substantially advanced understanding of brain organization, they often lacked the temporal resolution, cellular specificity, and reversible control necessary to determine whether activation of a particular neuronal population was sufficient or necessary for generating a specific physiological or behavioural response. The development of optogenetics fundamentally changed this paradigm by enabling selective optical activation or inhibition of genetically defined neurons with millisecond precision, thereby allowing functional interrogation of neural circuits *in vivo* and in real time (16, 19).

Neural circuits consist of highly organized networks of interconnected excitatory and inhibitory neurons that coordinate sensory processing, cognition, memory formation, emotional regulation, autonomic function, and motor behaviour. Disruption of these circuits underlies numerous neurological and psychiatric disorders, making precise mapping of neuronal connectivity essential for understanding disease pathogenesis. Optogenetics has emerged as the most powerful experimental platform for dissecting these complex networks because it permits selective manipulation of neuronal subpopulations based on neurotransmitter phenotype, genetic identity, developmental lineage, or projection targets while preserving the structural integrity of surrounding tissues (15).

Projection-specific optogenetics represents one of the most important advances in circuit neuroscience. Instead of stimulating neuronal cell bodies indiscriminately, optical fibers positioned over downstream target regions selectively activate axon terminals originating from genetically labelled neurons. This strategy has enabled unprecedented characterization of long-range corticothalamic, corticospinal, hippocampal, amygdalar, and basal ganglia projections, revealing previously unknown pathways involved in memory consolidation, emotional processing, motor coordination, and reward signalling (10). Combined with viral tracing technologies, including monosynaptic rabies virus tracing and trans-synaptic adeno-associated viral systems, optogenetics has facilitated comprehensive reconstruction of functional connectomes at cellular resolution (40).

Another major application involves functional interrogation of excitatory and inhibitory neuronal balance. Selective activation of glutamatergic pyramidal neurons or inhibition of *γ*-aminobutyric acid (GABA)-ergic interneurons has revealed how excitation–inhibition homeostasis governs cortical oscillations, sensory perception, attention, and decision-making. Conversely, optogenetic stimulation of parvalbumin-positive interneurons has demonstrated their central role in synchronizing gamma oscillations critical for cognitive processing and working memory. These findings have fundamentally reshaped understanding of cortical computation and highlighted neural circuit dysfunction as a central feature of numerous neurodegenerative and neuropsychiatric disorders (41, 42).

The integration of optogenetics with calcium imaging, functional magnetic resonance imaging (opto-fMRI), miniature microscopes, and high-density electrophysiological recordings has further expanded the ability to investigate dynamic neural networks across multiple spatial scales. Such multimodal approaches permit simultaneous manipulation and visualization of neuronal populations, providing comprehensive insight into information flow throughout distributed brain circuits. Consequently, optogenetic circuit mapping has become indispensable for identifying disease-associated neural pathways and validating therapeutic targets for neurological disorders (23).

### 4.2 Synaptic plasticity

Synaptic plasticity is widely regarded as the fundamental cellular mechanism underlying learning, memory, behavioural adaptation, and neural development. It refers to activity-dependent modifications in synaptic strength that alter the efficiency of communication between interconnected neurons. Two principal forms of synaptic plasticity, such as long-term potentiation (LTP) and long-term depression (LTD), have been extensively investigated as molecular substrates of information storage within the central nervous system. Optogenetics has revolutionized the study of these processes by enabling precise temporal control of presynaptic and postsynaptic neuronal activity, thereby allowing causal investigation of synaptic modifications with unprecedented precision (16, 43).

Conventional electrical stimulation indiscriminately activates heterogeneous neuronal populations and fibres of passage, making it difficult to isolate specific synaptic pathways responsible for plasticity. In contrast, optogenetics permits pathway-specific stimulation of genetically identified neuronal populations, enabling selective investigation of individual synapses within highly complex neural circuits. This capability has substantially improved understanding of Hebbian plasticity, spike timing-dependent plasticity (STDP), and homeostatic synaptic regulation. By delivering precisely timed optical pulses to presynaptic neurons while simultaneously recording postsynaptic responses, investigators have demonstrated how millisecond differences in spike timing determine whether synapses undergo potentiation or depression, providing direct experimental support for computational models of synaptic learning (15).

Optogenetic stimulation has significantly advanced understanding of molecular signalling pathways governing synaptic plasticity. Selective activation of hippocampal CA3–CA1 synapses has demonstrated that N-methyl-D-aspartate (NMDA) receptor-dependent calcium influx initiates intracellular signalling cascades involving calcium/calmodulin-dependent protein kinase II (CaMKII), protein kinase A (PKA), extracellular signal-regulated kinase (ERK), and cAMP response element-binding protein (CREB), ultimately leading to activity-dependent gene transcription and long-lasting synaptic strengthening (43). Conversely, prolonged low-frequency optical stimulation induces LTD through activation of protein phosphatases, AMPA receptor internalization, and alterations in dendritic spine morphology. These findings have provided mechanistic insight into how neuronal activity reshapes synaptic architecture during learning and memory formation.

Beyond excitatory synapses, optogenetics has transformed investigation of inhibitory plasticity mediated by GABAergic interneurons. Optical manipulation of parvalbumin-positive, somatostatin-positive, and vasoactive intestinal peptide (VIP)-expressing interneurons has demonstrated that inhibitory circuits actively regulate the threshold for LTP induction, cortical oscillations, and network synchronization rather than merely suppressing neuronal excitability. Such discoveries have fundamentally changed traditional concepts of synaptic integration by establishing inhibitory interneurons as dynamic regulators of information processing rather than passive modulators of circuit activity (41).

Recent advances combining optogenetics with super-resolution microscopy, genetically encoded calcium indicators, glutamate sensors, and single-spine imaging have enabled direct visualization of structural plasticity at individual synapses. Optical activation of defined neuronal ensembles has revealed rapid activity-dependent remodelling of dendritic spines, alterations in synaptic receptor trafficking, and localized protein synthesis that collectively contribute to long-term memory storage. Moreover, these technologies have demonstrated that synaptic plasticity is not confined to isolated neurons but instead emerges from coordinated interactions among neurons, astrocytes, microglia, and oligodendrocytes, highlighting the increasingly recognized importance of neuron–glia communication in regulating neural circuit function (44).

The application of optogenetics to models of neurodegenerative disease has further revealed that impaired synaptic plasticity frequently precedes overt neuronal degeneration. Experimental restoration of physiological activity patterns using optogenetic stimulation has partially rescued hippocampal LTP in Alzheimer's disease models, improved corticostriatal synaptic function in Parkinson's disease, and enhanced cortical reorganization following ischemic stroke. These findings suggest that synaptic dysfunction represents an early and potentially reversible therapeutic target, emphasizing the value of optogenetics for developing circuit-based interventions that extend beyond symptomatic treatment toward disease modification (16, 26).

### 4.3 Learning and memory

Learning and memory are among the most extensively investigated neurobiological processes using optogenetic approaches because they depend on highly coordinated neuronal ensembles distributed across multiple brain regions. Traditional behavioural, pharmacological, and electrophysiological studies established that the hippocampus, amygdala, prefrontal cortex, entorhinal cortex, and associated cortical structures collectively contribute to memory acquisition, consolidation, retrieval, and extinction. However, these approaches were unable to determine whether activation of specific neuronal populations was causally responsible for individual memory processes. The introduction of optogenetics fundamentally transformed this field by enabling selective activation or inhibition of genetically defined neuronal ensembles, commonly referred to as memory engrams, thereby providing direct evidence that discrete neuronal populations encode specific memories (16, 45).

The concept of memory engrams originated more than a century ago but remained experimentally inaccessible until the development of activity-dependent genetic labeling strategies combined with optogenetic manipulation. Using immediate-early gene promoters such as *c-fos* and *Arc*, neurons activated during memory formation can be permanently labelled with excitatory opsins such as Channelrhodopsin-2 (ChR2). Subsequent optical stimulation of these labelled neurons has demonstrated that artificial reactivation of engram cells is sufficient to evoke behavioural recall even in the absence of the original environmental cues. Conversely, selective optical inhibition of the same neuronal population prevents memory retrieval without affecting unrelated memories, thereby establishing the causal relationship between neuronal ensemble activity and memory expression (46, 47). These pioneering studies fundamentally changed classical theories of memory storage by demonstrating that memories are encoded within sparse yet highly organized neuronal ensembles rather than diffusely distributed neuronal networks.

Optogenetic approaches have also substantially advanced understanding of the cellular mechanisms governing synaptic modifications during memory formation. Selective stimulation of hippocampal CA3–CA1 pathways has revealed how temporally coordinated neuronal firing induces long-term potentiation (LTP), strengthens synaptic efficacy, and promotes activity-dependent gene expression through signaling pathways involving calcium/calmodulin-dependent protein kinase II (CaMKII), cAMP response element-binding protein (CREB), and brain-derived neurotrophic factor (BDNF). Conversely, prolonged optical inhibition induces long-term depression (LTD), facilitating synaptic weakening and memory updating. These findings demonstrate that learning reflects dynamic modulation of synaptic connectivity rather than static structural changes, emphasizing the remarkable plasticity of neuronal circuits throughout life (43, 45).

Beyond declarative memory, optogenetics has significantly expanded understanding of emotional learning and fear conditioning. The amygdala serves as a central hub integrating sensory inputs with emotional significance, particularly during fear acquisition and extinction. Selective activation of basolateral amygdala projections to the central amygdala has been shown to enhance conditioned fear responses, whereas optical inhibition of the same pathways suppresses fear expression and facilitates extinction learning. These observations have identified previously unrecognized neural circuits responsible for anxiety disorders and post-traumatic stress disorder (PTSD), providing potential therapeutic targets for circuit-based interventions (10). Similarly, optogenetic manipulation of prefrontal cortical projections has demonstrated their essential role in executive control of memory retrieval, decision-making, and cognitive flexibility, highlighting the distributed nature of higher-order cognitive processing.

One of the most remarkable achievements of optogenetics has been the demonstration that impaired memory function associated with neurodegenerative diseases may be partially reversible through targeted neural circuit modulation. In transgenic mouse models of Alzheimer's disease, selective optical activation of hippocampal engram cells restored memory retrieval despite persistent amyloid pathology, suggesting that memory loss may initially arise from impaired access to stored information rather than irreversible erasure of memory traces (26). Furthermore, optogenetic induction of gamma-frequency oscillations within hippocampal and cortical circuits has been shown to reduce amyloid burden, enhance microglial phagocytosis, and improve cognitive performance, revealing an unexpected relationship between neuronal oscillations and neuroimmune regulation (27). Collectively, these discoveries have transformed understanding of memory biology and established optogenetics as an indispensable platform for investigating cognitive dysfunction and developing precision therapies for neurodegenerative disorders.

### 4.4 Sensory and motor systems

The nervous system continuously integrates sensory information from the external environment to generate coordinated motor responses essential for survival. Sensory perception and motor control emerge from highly organized neuronal circuits distributed throughout the cerebral cortex, thalamus, brainstem, spinal cord, cerebellum, and peripheral nervous system. Owing to their anatomical complexity and rapid temporal dynamics, these systems have historically been difficult to investigate using conventional experimental techniques. Optogenetics has revolutionized sensory and motor neuroscience by enabling selective manipulation of genetically defined neuronal populations with millisecond precision, thereby providing direct insights into how neural circuits transform sensory inputs into behavioural outputs (16, 23).

Within the visual system, optogenetics has provided unprecedented opportunities to investigate retinal signal processing, visual cortex organization, and higher-order perception. Selective activation of retinal ganglion cells, bipolar cells, and photoreceptor pathways has facilitated detailed analysis of visual information processing while simultaneously providing the foundation for experimental vision restoration therapies in retinal degenerative diseases. Indeed, optogenetic retinal stimulation has progressed into early-phase clinical studies for inherited retinal disorders, demonstrating the translational potential of optical neuromodulation for sensory rehabilitation (48). Similar strategies have been employed in auditory neuroscience, where optical stimulation of cochlear spiral ganglion neurons has achieved improved temporal fidelity compared with conventional electrical cochlear implants, suggesting future applications in high-resolution auditory prostheses.

Somatosensory processing has likewise benefited substantially from optogenetic methodologies. Selective manipulation of mechanoreceptors, nociceptors, proprioceptors, and spinal dorsal horn neurons has enabled detailed characterization of neural pathways underlying touch, temperature sensation, itch, and pain perception. Optical activation of nociceptive afferents reliably evokes pain-related behaviours, whereas inhibition of the same neuronal populations suppresses mechanical and thermal hypersensitivity in experimental models of chronic neuropathic pain. These studies have provided compelling evidence that chronic pain results from maladaptive circuit plasticity rather than solely peripheral tissue injury, thereby identifying novel therapeutic opportunities for circuit-specific neuromodulation (49).

Motor control represents another major area in which optogenetics has fundamentally transformed systems neuroscience. Coordinated voluntary movement requires precise interactions among corticospinal neurons, basal ganglia, cerebellar circuits, brainstem nuclei, and spinal motor networks. Conventional electrical stimulation indiscriminately activates multiple neuronal populations, making it difficult to distinguish individual circuit contributions. In contrast, optogenetics enables selective activation of corticospinal neurons, medium spiny neurons of the striatum, cerebellar Purkinje cells, or spinal interneurons, thereby revealing their distinct functional roles in movement initiation, coordination, balance, and motor learning (50). These investigations have significantly refined contemporary models of basal ganglia function and clarified how direct and indirect pathways interact to regulate voluntary movement.

Optogenetic manipulation has also transformed understanding of motor disorders associated with neurodegeneration. Selective stimulation of dopaminergic neurons within the substantia nigra pars compacta or modulation of direct pathway medium spiny neurons has improved motor deficits in experimental models of Parkinson's disease while simultaneously validating mechanisms underlying deep-brain stimulation. Likewise, optical stimulation of corticospinal circuits following spinal cord injury and ischemic stroke has promoted axonal sprouting, cortical reorganization, and functional recovery, highlighting the remarkable capacity of adult neural circuits for activity-dependent plasticity (28). These findings support the concept that restoration of physiological neuronal activity may represent an effective therapeutic strategy for motor rehabilitation.

Recent technological advances have further expanded the capabilities of sensory and motor circuit research through integration of optogenetics with high-density electrophysiology, miniature microscopes, wearable wireless photonic devices, functional ultrasound imaging, and artificial intelligence-assisted behavioural analysis. Such multimodal approaches permit simultaneous manipulation and monitoring of distributed neuronal populations during freely moving behaviour, providing unprecedented insight into neural computation underlying perception, movement, and adaptive behaviour. Consequently, optogenetics has become an indispensable methodology for investigating the fundamental principles governing sensorimotor integration while simultaneously accelerating development of next-generation neuroprosthetics, brain–computer interfaces, and precision neuromodulation therapies for neurological disease.

### 4.5 Neurodevelopment

Neurodevelopment is a highly coordinated and dynamic biological process that encompasses the generation, migration, differentiation, maturation, and functional integration of neurons and glial cells into complex neural circuits. These tightly regulated events begin during embryogenesis and continue throughout postnatal life, ultimately establishing the structural and functional architecture of the central nervous system. Precise regulation of neurodevelopment is essential for normal cognitive, sensory, motor, and behavioural functions, whereas disturbances in these processes contribute to numerous neurological and neurodevelopmental disorders, including autism spectrum disorder (ASD), schizophrenia, intellectual disability, epilepsy, and cerebral palsy (51, 52). Although classical developmental biology and genetic approaches have identified many of the molecular pathways governing nervous system development, they have been unable to establish direct causal relationships between neuronal activity and developmental circuit assembly. Optogenetics has overcome this limitation by providing reversible, temporally precise, and cell-specific control of neuronal excitability during defined developmental windows, thereby enabling unprecedented investigation of activity-dependent mechanisms underlying brain maturation (16).

Neuronal activity is now recognized as a fundamental regulator of neurodevelopment rather than merely a consequence of mature circuit formation. During embryonic and early postnatal development, spontaneous electrical activity emerges before the maturation of sensory systems and serves as an intrinsic instructive signal guiding neuronal differentiation, axonal extension, dendritic arborization, synaptogenesis, and refinement of neuronal connectivity. Optogenetic manipulation has enabled selective enhancement or suppression of this spontaneous activity in genetically defined neuronal populations, revealing that even subtle alterations in firing frequency can profoundly influence neuronal morphology, migration patterns, and circuit organization (15). These observations have established neuronal activity as an essential epigenetic regulator that interacts with genetic and environmental cues to shape brain development (53).

One of the earliest applications of optogenetics in developmental neuroscience focused on neuronal migration within the developing cerebral cortex. Cortical projection neurons are generated in the ventricular zone before undergoing radial migration toward their final laminar positions. Optical modulation of immature cortical neurons has demonstrated that membrane depolarization regulates intracellular calcium oscillations, cytoskeletal dynamics, and guidance signaling pathways, thereby influencing migration velocity and positioning within cortical layers (54). Conversely, prolonged inhibition of neuronal activity during critical developmental periods disrupts cortical lamination and alters subsequent synaptic connectivity, highlighting the importance of electrical activity in establishing functional cortical architecture.

Optogenetics has also substantially advanced understanding of axonal pathfinding and target recognition. During neural development, extending axons navigate through complex extracellular environments using molecular guidance cues such as netrins, semaphorins, ephrins, and Slit proteins. Increasing evidence indicates that neuronal activity interacts with these molecular guidance systems to refine axonal trajectories and stabilize appropriate synaptic contacts. Selective optical stimulation of developing neurons has revealed that localized calcium influx regulates growth cone motility, actin polymerization, and microtubule organization, thereby directing axonal extension toward appropriate targets. Furthermore, optogenetic manipulation has demonstrated that activity-dependent competition between neighbouring axons contributes significantly to the refinement of sensory and motor circuits during postnatal maturation (55).

The formation and maturation of synaptic connections represent another critical aspect of neurodevelopment that has been profoundly illuminated by optogenetic methodologies. Synaptogenesis involves the coordinated assembly of presynaptic release machinery, postsynaptic receptor complexes, and specialized scaffolding proteins, followed by selective elimination of redundant connections during activity-dependent synaptic pruning. Optical stimulation of immature neuronal networks has demonstrated that patterned neuronal activity promotes dendritic spine formation, enhances excitatory synapse maturation, and accelerates functional integration of newly generated neurons into existing circuits. Conversely, chronic suppression of neuronal firing impairs synaptic stabilization and disrupts excitatory-inhibitory balance, emphasizing the essential role of neuronal activity in sculpting mature neural networks (16, 43).

An important contribution of optogenetics has been its ability to investigate developmental critical periods, during which neural circuits exhibit heightened sensitivity to environmental and sensory experiences. Classical studies suggested that sensory deprivation permanently alters cortical organization; however, optogenetic manipulation has revealed that precise modulation of neuronal activity can reopen or extend critical periods, thereby enhancing neuronal plasticity even during adulthood. This discovery has important implications for neurorehabilitation, suggesting that activity-dependent circuit remodeling may be therapeutically harnessed following developmental injury or neurological disease. Moreover, optogenetic stimulation has identified molecular mediators of critical period plasticity, including brain-derived neurotrophic factor (BDNF), N-methyl-D-aspartate (NMDA) receptor signaling, and parvalbumin-positive interneuron maturation, thereby providing mechanistic insights into developmental circuit refinement (56, 57).

Recent advances have further expanded the role of optogenetics in developmental neuroscience through integration with stem cell biology, cerebral organoid technology, and single-cell multi-omics. Human induced pluripotent stem cell (iPSC)-derived neurons expressing microbial opsins permit functional characterization of developing neuronal networks *in vitro*, enabling investigation of patient-specific developmental abnormalities associated with autism spectrum disorder, Rett syndrome, fragile X syndrome, and other genetic neurodevelopmental conditions. Similarly, optogenetic interrogation of three-dimensional cerebral organoids has facilitated direct examination of emerging neuronal connectivity, oscillatory activity, and synaptic maturation in human brain-like tissues, providing valuable experimental platforms for modelling early neurodevelopment and evaluating therapeutic interventions (58).

Optogenetics has also significantly enhanced understanding of neuron–glia interactions during nervous system development. Astrocytes, oligodendrocytes, and microglia actively participate in synapse formation, axonal myelination, metabolic support, and immune surveillance throughout brain maturation. Optical manipulation of glial calcium signaling has demonstrated that astrocytic activity regulates neuronal excitability and synaptic maturation through gliotransmitter release, whereas optogenetic studies of microglia have revealed their essential role in activity-dependent synaptic pruning and refinement of neuronal connectivity. Furthermore, emerging evidence indicates that oligodendrocyte precursor cells respond dynamically to neuronal activity, adjusting myelination patterns to optimize action potential conduction and network synchronization during development (59, 60). These findings have substantially broadened traditional neuron-centric models of neurodevelopment by highlighting the integrated contributions of multiple cellular populations to functional circuit assembly.

From a translational perspective, optogenetics has become an invaluable platform for investigating developmental disorders characterized by aberrant neuronal connectivity and impaired circuit maturation. Experimental modulation of excitatory and inhibitory neuronal balance has reproduced key pathological features of autism spectrum disorder and schizophrenia, whereas restoration of physiological neuronal activity has partially rescued behavioural deficits in several animal models. Such observations suggest that many neurodevelopmental disorders arise not solely from irreversible structural abnormalities but also from dysfunctional neuronal activity patterns that may remain amenable to therapeutic modulation. Consequently, optogenetics provides an unprecedented opportunity to identify critical developmental windows during which targeted neuromodulation could modify disease progression and improve long-term neurological outcomes.

### 4.6 Sleep and circadian regulation

Sleep and circadian rhythms are fundamental physiological processes that regulate numerous aspects of brain function, including neuronal excitability, synaptic plasticity, memory consolidation, energy metabolism, immune responses, and metabolic homeostasis. The sleep-wake cycle is governed by an intricate network of neuronal populations distributed across the hypothalamus, brainstem, thalamus, basal forebrain, and cerebral cortex, while circadian rhythmicity is orchestrated by the suprachiasmatic nucleus (SCN) of the hypothalamus, which serves as the master biological clock. Disruption of sleep architecture and circadian rhythms is increasingly recognized as both a consequence and a contributing factor in the pathogenesis of several neurodegenerative disorders, including Alzheimer's disease, Parkinson's disease, Huntington's disease, and amyotrophic lateral sclerosis (61, 62). Optogenetics has revolutionized this field by enabling reversible, cell-type-specific manipulation of sleep-regulating neuronal circuits with millisecond precision, thereby providing direct mechanistic insights into the neural substrates governing sleep and circadian behaviour.

Traditional approaches to sleep research, including electroencephalography (EEG), pharmacological interventions, and lesion studies, have identified many of the brain regions involved in sleep regulation but have been unable to establish causal relationships between specific neuronal populations and behavioural state transitions. Optogenetics has overcome these limitations by permitting selective activation or inhibition of genetically defined neurons during natural sleep and wakefulness. Optical stimulation of hypocretin (orexin)-expressing neurons within the lateral hypothalamus has demonstrated their pivotal role in promoting wakefulness and stabilizing arousal states, whereas inhibition of these neurons induces rapid transitions into non-rapid eye movement (NREM) sleep. Conversely, activation of melanin-concentrating hormone (MCH) neurons facilitates REM sleep and contributes to sleep-dependent memory processing, illustrating the complementary functions of neighbouring hypothalamic neuronal populations in regulating behavioural states (63, 64).

Optogenetic investigations have also identified several additional neuronal populations that collectively regulate sleep architecture. GABAergic neurons of the ventrolateral preoptic nucleus (VLPO) actively suppress wake-promoting centres to initiate and maintain sleep, while cholinergic neurons of the basal forebrain and brainstem contribute to cortical activation during wakefulness and REM sleep. Likewise, glutamatergic neurons within the parabrachial nucleus, histaminergic neurons of the tuberomammillary nucleus, serotonergic neurons of the dorsal raphe, and noradrenergic neurons of the locus coeruleus interact dynamically to regulate vigilance, arousal, and transitions between sleep stages. Optogenetic manipulation of these circuits has provided direct evidence supporting the “flip-flop switch” model of sleep regulation, in which reciprocal inhibition between sleep-promoting and wake-promoting neuronal populations ensures rapid and stable transitions between behavioural states (61, 65).

Beyond regulating sleep architecture, optogenetics has substantially advanced understanding of the relationship between sleep and memory consolidation. During NREM sleep, synchronized cortical slow oscillations, thalamocortical sleep spindles, and hippocampal sharp-wave ripples coordinate the transfer of newly acquired information from temporary hippocampal storage to long-term cortical memory networks. Optogenetic modulation of these oscillatory events has demonstrated that selective enhancement of hippocampal sharp-wave ripples or cortical slow oscillations significantly improves memory consolidation, whereas disruption of these rhythms impairs learning and cognitive performance (66). These findings provide compelling evidence that sleep is an active neurophysiological process essential for synaptic remodeling, neuronal plasticity, and cognitive function rather than a passive period of reduced brain activity.

Circadian regulation has similarly benefited from optogenetic approaches. The SCN receives photic information from intrinsically photosensitive retinal ganglion cells expressing melanopsin and synchronizes peripheral biological clocks through coordinated neuronal and hormonal signaling. Optogenetic stimulation of SCN neurons has demonstrated that neuronal firing frequency directly influences circadian phase resetting, gene expression, and behavioural rhythmicity. Furthermore, selective manipulation of vasoactive intestinal peptide (VIP)-expressing and arginine vasopressin (AVP)-expressing SCN neurons has revealed their distinct contributions to circadian synchronization and network stability. These studies have significantly refined current models of circadian pacemaker organization by demonstrating that coordinated activity among genetically defined neuronal subpopulations is essential for maintaining robust circadian rhythms (67).

Sleep disturbances are among the earliest clinical manifestations of many neurodegenerative diseases, often preceding overt cognitive or motor impairment by several years. Increasing evidence indicates that disrupted sleep accelerates neurodegeneration through impaired glymphatic clearance of neurotoxic proteins, enhanced oxidative stress, chronic neuroinflammation, and altered synaptic homeostasis. Optogenetic studies have demonstrated that restoring physiological sleep patterns or enhancing specific neuronal oscillations improves glymphatic clearance of *β*-amyloid, reduces tau accumulation, attenuates neuroinflammatory responses, and improves cognitive performance in experimental models of Alzheimer's disease (62). Similarly, modulation of hypothalamic and brainstem sleep circuits has alleviated sleep fragmentation and motor dysfunction in models of Parkinson's disease, highlighting the therapeutic potential of circuit-specific neuromodulation for neurodegenerative disorders.

Recent technological advances have further expanded the application of optogenetics in sleep research through integration with wireless photonic devices, miniature EEG recording systems, fibre photometry, calcium imaging, and closed-loop neuromodulation. These multimodal approaches permit simultaneous monitoring and manipulation of neuronal activity in freely behaving animals across multiple sleep cycles, enabling precise characterization of neuronal dynamics underlying sleep initiation, maintenance, and awakening. Moreover, coupling optogenetics with transcriptomics, proteomics, and artificial intelligence-based behavioural analysis has provided comprehensive insights into the molecular and circuit-level mechanisms linking sleep, circadian regulation, and neurological health (23). Collectively, these advances have transformed sleep neuroscience from a predominantly descriptive discipline into a mechanistic science capable of identifying causal neural circuits and novel therapeutic targets. As sleep dysfunction is increasingly recognized as both a biomarker and modifiable risk factor for neurodegenerative diseases, optogenetics continues to provide a powerful experimental framework for understanding the bidirectional relationship between neuronal circuit activity, circadian regulation, and brain health.

## 5 Optogenetics for understanding neurodegenerative disease mechanisms

Neurodegenerative disorders are characterized by progressive neuronal dysfunction and irreversible loss of specific neuronal populations, ultimately leading to cognitive impairment, motor deficits, and behavioural abnormalities. Although individual diseases exhibit distinct pathological signatures, accumulating evidence indicates that they converge on a limited number of interconnected cellular and molecular mechanisms, including abnormal protein aggregation, synaptic dysfunction, chronic neuroinflammation, mitochondrial impairment, oxidative stress, and regulated cell death. These pathological processes interact dynamically within neural circuits, progressively disrupting neuronal communication and network integrity long before extensive neuronal loss becomes clinically apparent (2, 3). The major cellular and molecular mechanisms investigated using optogenetics are summarized in Figure 3, illustrating how circuit-specific optical modulation has advanced mechanistic understanding of neurodegenerative disease pathogenesis.

**Figure 3:**
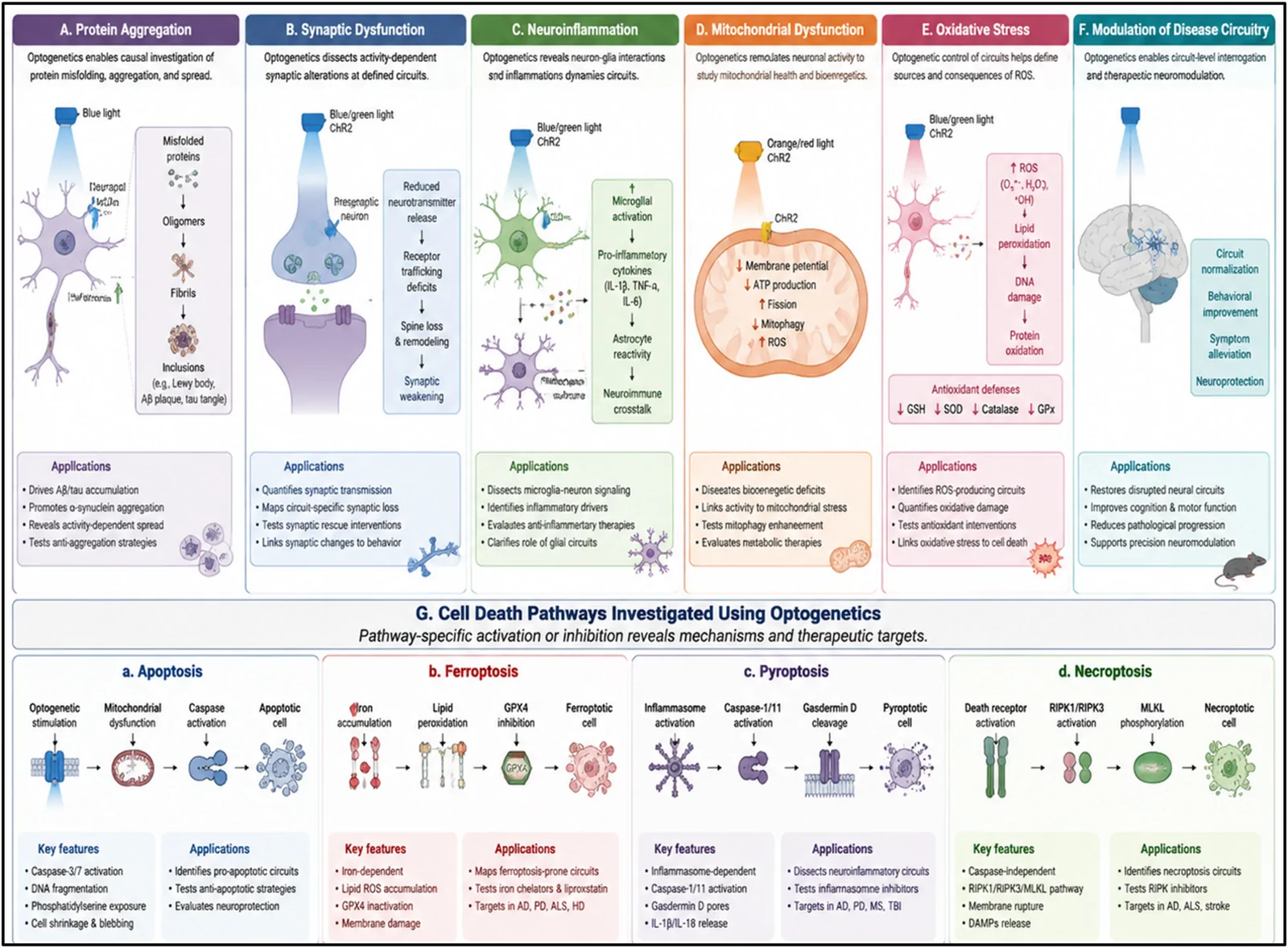
Cellular mechanisms investigated using optogenetics in neurodegenerative disorders. Optogenetics enables precise manipulation of genetically defined neuronal populations to investigate key pathological mechanisms underlying neurodegeneration. Optical modulation has provided mechanistic insights into protein aggregation **(A)**, synaptic dysfunction **(B)**, neuroinflammation **(C)**, mitochondrial impairment **(D)**, oxidative stress **(E)**, and regulated cell death pathways including apoptosis, ferroptosis, pyroptosis, and necroptosis **(F)** Collectively, these approaches establish causal links between cellular pathology, neural circuit dysfunction, and disease progression, facilitating the identification of novel therapeutic targets for precision neuromodulation **(G)**.

Traditional experimental approaches have successfully identified numerous molecular abnormalities associated with neurodegeneration but frequently fail to establish direct causal relationships between cellular pathology and circuit dysfunction. Optogenetics has fundamentally transformed this field by enabling precise manipulation of genetically defined neuronal populations during disease progression. By selectively activating or inhibiting vulnerable neuronal circuits, investigators can determine whether specific pathological mechanisms directly impair neuronal communication or arise as secondary consequences of ongoing neurodegeneration. Furthermore, optogenetic interrogation of disease-associated neural networks has revealed that many pathological changes initially manifest as functional disturbances within neural circuits before overt neuronal death occurs, emphasizing the importance of circuit-level analysis for early disease detection and therapeutic intervention (10, 16).

### 5.1 Protein aggregation

Aberrant protein aggregation is a defining pathological hallmark shared by most neurodegenerative diseases and represents one of the earliest molecular events contributing to neuronal dysfunction. Progressive accumulation of misfolded proteins, including amyloid-*β* (A*β*) and hyperphosphorylated tau in Alzheimer's disease, *α*-synuclein in Parkinson's disease, mutant huntingtin in Huntington's disease, and TAR DNA-binding protein-43 (TDP-43) in amyotrophic lateral sclerosis disrupts intracellular homeostasis, impairs synaptic communication, and initiates widespread neurodegenerative cascades (11, 68). Increasing evidence suggests that these aggregates propagate through interconnected neuronal networks in a prion-like manner, facilitating disease progression across anatomically connected brain regions.

Optogenetics has emerged as a powerful platform for investigating how protein aggregates influence neuronal circuit function. Selective optical stimulation of vulnerable neuronal populations has demonstrated that pathological protein accumulation initially alters neuronal excitability and synaptic transmission before causing irreversible structural degeneration. In transgenic Alzheimer's disease models, optogenetic activation of hippocampal engram cells restored memory retrieval despite persistent amyloid deposition, indicating that early cognitive impairment may result primarily from circuit dysfunction rather than complete neuronal loss (26). Likewise, gamma-frequency (40 Hz) optogenetic stimulation of cortical and hippocampal interneurons has significantly reduced amyloid burden by enhancing microglial phagocytosis and improving glymphatic clearance of extracellular protein aggregates (27). These findings have fundamentally altered conventional concepts of amyloid pathology by demonstrating that neuronal activity itself influences protein aggregation dynamics and innate immune responses.

In synucleinopathies, optogenetic interrogation of dopaminergic circuits has revealed that *α*-synuclein accumulation disrupts basal ganglia network synchronization and alters neuronal firing patterns long before degeneration of substantia nigra neurons becomes extensive. Similar observations have been reported in Huntington's disease, where mutant huntingtin selectively impairs corticostriatal connectivity and disrupts neuronal oscillations underlying voluntary movement. Collectively, these studies suggest that abnormal protein aggregation should be viewed not merely as a static histopathological feature but as a dynamic regulator of neural circuit dysfunction that may remain therapeutically reversible during early disease stages (12).

### 5.2 Synaptic dysfunction

Synaptic dysfunction is increasingly recognized as one of the earliest and most important pathological mechanisms underlying neurodegeneration. Long before widespread neuronal death becomes apparent, abnormalities in neurotransmitter release, dendritic spine morphology, receptor trafficking, and synaptic plasticity progressively compromise neuronal communication, ultimately disrupting higher-order cognitive and motor functions (11, 69, 70). Consequently, synaptic failure has emerged as a stronger predictor of clinical disease severity than the absolute extent of neuronal loss in several neurodegenerative disorders.

Optogenetics has transformed understanding of synaptic pathology by enabling selective stimulation of defined neuronal pathways while simultaneously recording electrophysiological responses at downstream synapses. Such pathway-specific interrogation has revealed that amyloid-*β* oligomers preferentially impair long-term potentiation (LTP), facilitate long-term depression (LTD), and reduce dendritic spine density within hippocampal circuits responsible for learning and memory. Importantly, optogenetic restoration of physiological neuronal activity partially rescues synaptic plasticity and improves cognitive performance in several experimental Alzheimer's disease models, highlighting synaptic dysfunction as a potentially reversible therapeutic target (26).

Similarly, optogenetic studies have demonstrated that dopaminergic depletion in Parkinson's disease profoundly alters corticostriatal synaptic transmission by disrupting the balance between direct and indirect basal ganglia pathways. Selective activation of direct-pathway medium spiny neurons restores physiological motor output, whereas stimulation of indirect-pathway neurons exaggerates motor impairment, thereby providing definitive evidence supporting classical basal ganglia circuit models (50). In Huntington's disease, optical interrogation has revealed early impairment of corticostriatal glutamatergic transmission before overt neuronal degeneration, suggesting that dysfunctional synaptic communication precedes structural pathology.

Recent integration of optogenetics with super-resolution microscopy, genetically encoded calcium indicators, glutamate sensors, and single-synapse imaging has enabled direct visualization of activity-dependent receptor trafficking, neurotransmitter release, and dendritic spine remodeling. These multimodal approaches have demonstrated that synaptic dysfunction reflects complex interactions among neurons, astrocytes, and microglia rather than isolated neuronal abnormalities alone, emphasizing the importance of multicellular circuit interactions in neurodegenerative disease progression (59).

### 5.3 Neuroinflammation

Neuroinflammation has emerged as a central driver of neurodegenerative disease progression rather than merely a secondary response to neuronal injury. Chronic activation of microglia and astrocytes promotes sustained production of pro-inflammatory cytokines, reactive oxygen species, complement proteins, and chemokines that progressively disrupt synaptic function, impair neuronal metabolism, and accelerate neuronal degeneration (9, 71). Although acute inflammatory responses may initially facilitate tissue repair and protein clearance, persistent neuroinflammation establishes a self-perpetuating cycle of neuronal injury and immune activation that contributes significantly to disease progression.

Optogenetics has provided unprecedented opportunities to investigate bidirectional communication between neurons and glial cells during neuroinflammation. Selective activation of neuronal circuits has demonstrated that neuronal firing patterns directly regulate microglial morphology, motility, and phagocytic activity through activity-dependent release of neurotransmitters, ATP, and chemokines. Conversely, optical manipulation of astrocytic calcium signaling has revealed that astrocytes actively modulate neuronal excitability, synaptic plasticity, and neurovascular coupling through gliotransmitter release rather than functioning solely as passive structural support cells (59).

One of the most influential discoveries in recent years has been the demonstration that optogenetically induced gamma oscillations significantly modify innate immune responses in Alzheimer's disease. Gamma-frequency stimulation activates parvalbumin-positive interneurons, resulting in enhanced microglial recruitment, increased phagocytosis of amyloid plaques, reduced neuroinflammatory cytokine production, and improved cognitive function. These findings have established a direct mechanistic link between neuronal oscillatory activity and immune regulation within the central nervous system, providing a novel therapeutic paradigm in which modulation of neural circuit activity indirectly influences neuroimmune function (27).

Furthermore, optogenetic interrogation of hypothalamic and brainstem inflammatory circuits has demonstrated that neuronal activity regulates systemic immune responses through autonomic pathways, illustrating the extensive integration between the nervous and immune systems. Combined with single-cell transcriptomics and spatial transcriptomic technologies, optogenetics now enables precise characterization of disease-associated microglial and astrocytic phenotypes within anatomically defined neural circuits. These multidisciplinary approaches are rapidly transforming understanding of neuroinflammation from a generalized pathological process into a circuit-specific and cell-specific mechanism that contributes directly to neurodegenerative disease progression (9).

### 5.4 Mitochondrial dysfunction

Mitochondria are indispensable organelles that regulate cellular energy metabolism, calcium homeostasis, redox signaling, biosynthetic pathways, and programmed cell death. Given the exceptionally high metabolic demands of neurons, maintenance of mitochondrial integrity is essential for sustaining synaptic transmission, axonal transport, and neuronal survival. Consequently, mitochondrial dysfunction has emerged as a central pathogenic mechanism shared by virtually all major neurodegenerative disorders, including Alzheimer's disease, Parkinson's disease, Huntington's disease and amyotrophic lateral sclerosis (72, 73). Accumulating evidence indicates that mitochondrial abnormalities occur during the earliest stages of disease progression, preceding extensive neuronal loss and contributing directly to synaptic impairment, oxidative stress, neuroinflammation, and activation of multiple cell death pathways.

Healthy neurons rely on a dynamic mitochondrial network maintained through coordinated processes of mitochondrial biogenesis, fusion, fission, intracellular transport, and selective autophagic degradation (mitophagy). Disruption of these quality-control mechanisms results in defective oxidative phosphorylation, impaired ATP production, excessive reactive oxygen species (ROS) generation, calcium dysregulation, and reduced metabolic flexibility. In Alzheimer's disease, amyloid-*β* and hyperphosphorylated tau interfere with mitochondrial respiratory complexes, impair mitochondrial trafficking along axons, and inhibit mitophagy, thereby compromising neuronal bioenergetics and accelerating synaptic degeneration (73). Similarly, mutations in genes such as *PINK1*, *Parkin*, *DJ-1*, and *LRRK2* disrupt mitochondrial quality-control pathways in Parkinson's disease, leading to accumulation of dysfunctional mitochondria within dopaminergic neurons of the substantia nigra and increasing their susceptibility to degeneration (74).

Optogenetics has provided unprecedented opportunities to investigate the causal relationship between mitochondrial dysfunction and neuronal circuit activity. Unlike conventional biochemical analyses, which primarily assess mitochondrial abnormalities in isolated tissues or cultured cells, optogenetic approaches enable selective modulation of disease-relevant neuronal populations while simultaneously monitoring mitochondrial function in intact neural circuits. Optical activation of genetically defined neurons combined with genetically encoded fluorescent biosensors for ATP, calcium, mitochondrial membrane potential, and ROS has revealed that neuronal firing rapidly influences mitochondrial dynamics and metabolic activity. These studies demonstrate that mitochondrial function is not static but is continuously regulated by neuronal activity, synaptic demand, and intracellular calcium signaling (23).

Recent advances have further expanded the application of optogenetics through the development of optogenetic control of mitochondrial function, commonly referred to as mito-optogenetics. In these systems, light-sensitive proteins are selectively targeted to mitochondrial membranes, allowing investigators to manipulate mitochondrial membrane potential, proton gradients, calcium uptake, or reactive oxygen species production with high spatial and temporal precision. Such approaches have enabled direct investigation of how transient mitochondrial depolarization influences neuronal excitability, synaptic plasticity, and metabolic adaptation without the confounding off-target effects associated with pharmacological mitochondrial inhibitors (75). Furthermore, optogenetic regulation of mitochondrial dynamics has demonstrated that restoration of physiological mitochondrial function can improve neuronal survival and partially rescue synaptic deficits in experimental neurodegenerative disease models.

Another major contribution of optogenetics has been elucidating the relationship between mitochondrial dysfunction and neuronal network activity. Hyperactive neuronal circuits impose substantial energetic demands that increase ATP consumption and calcium influx, thereby exacerbating mitochondrial stress and ROS production. Conversely, prolonged neuronal hypoactivity reduces mitochondrial biogenesis and impairs metabolic adaptability. Optogenetic manipulation of hippocampal and cortical circuits has demonstrated that restoring physiological patterns of neuronal activity normalizes mitochondrial calcium handling, improves respiratory efficiency, and enhances neuronal resilience against metabolic stress. These observations suggest that neuronal activity itself is an important determinant of mitochondrial health and that circuit-level interventions may indirectly improve mitochondrial function during neurodegeneration (16).

Mitochondrial dysfunction also interacts closely with neuroinflammation and protein aggregation, creating a self-amplifying pathogenic cycle. Damaged mitochondria release mitochondrial DNA, cytochrome *c*, cardiolipin, and other damage-associated molecular patterns (DAMPs) that activate innate immune signaling and inflammasome pathways. Concurrently, impaired ATP synthesis and excessive ROS production promote protein misfolding, synaptic dysfunction, and activation of apoptotic and ferroptotic pathways. Optogenetic studies have shown that modulation of neuronal firing patterns can influence these interconnected processes by improving mitochondrial quality control, reducing metabolic stress, and preserving neuronal circuit integrity. Consequently, mitochondrial dysfunction is now recognized not only as a metabolic abnormality but also as a critical regulator of neuronal communication and disease progression.

### 5.5 Oxidative stress

Oxidative stress is a fundamental pathological mechanism underlying the initiation and progression of numerous neurodegenerative disorders. It results from an imbalance between the generation of reactive oxygen species (ROS) and reactive nitrogen species (RNS) and the capacity of endogenous antioxidant defense systems to neutralize these reactive molecules. Owing to their exceptionally high oxygen consumption, abundant polyunsaturated fatty acids, relatively low antioxidant capacity, and dependence on mitochondrial oxidative phosphorylation, neurons are particularly susceptible to oxidative damage. Persistent oxidative stress disrupts cellular homeostasis by damaging lipids, proteins, nucleic acids, and organelles, ultimately leading to synaptic dysfunction, impaired neuronal communication, mitochondrial failure, and progressive neurodegeneration (76, 77).

The principal intracellular sources of ROS include the mitochondrial electron transport chain, nicotinamide adenine dinucleotide phosphate (NADPH) oxidases (NOXs), xanthine oxidase, uncoupled nitric oxide synthase (NOS), and activated microglia. Under physiological conditions, low concentrations of ROS serve as essential signaling molecules regulating neuronal differentiation, synaptic plasticity, calcium homeostasis, and gene expression. However, excessive ROS production overwhelms antioxidant defense systems, including superoxide dismutase (SOD), catalase (CAT), glutathione peroxidase (GPx), glutathione (GSH), thioredoxin, and peroxiredoxins, resulting in oxidative damage to critical cellular components (76). Oxidative stress therefore represents not merely a consequence of neurodegeneration but also a major driving force that amplifies disease progression through multiple interconnected molecular pathways.

Substantial evidence indicates that oxidative stress is an early pathological event in Alzheimer's disease. Soluble amyloid-*β* oligomers promote excessive ROS production by impairing mitochondrial respiration, activating NOX enzymes, and disturbing intracellular calcium homeostasis. Simultaneously, hyperphosphorylated tau further aggravates oxidative injury by disrupting axonal transport of mitochondria and reducing neuronal bioenergetic efficiency. The resulting oxidative damage promotes lipid peroxidation, protein carbonylation, DNA oxidation, and impairment of synaptic signaling, ultimately contributing to cognitive decline and neuronal loss (78). Similarly, degeneration of dopaminergic neurons in Parkinson's disease is strongly associated with oxidative metabolism of dopamine, mitochondrial complex I dysfunction, iron accumulation, and neuroinflammatory activation, all of which synergistically increase ROS generation and accelerate neuronal degeneration (79).

Optogenetics has significantly advanced understanding of the relationship between neuronal activity and oxidative stress by enabling precise manipulation of disease-relevant neural circuits while simultaneously monitoring intracellular redox dynamics. Conventional biochemical approaches generally measure oxidative damage at the tissue level and therefore fail to capture the rapid temporal changes occurring within individual neurons or specific neural circuits. In contrast, optogenetic stimulation combined with genetically encoded fluorescent biosensors for hydrogen peroxide, glutathione, and redox-sensitive green fluorescent protein (roGFP) enables real-time visualization of oxidative responses in living neurons during physiological and pathological activity (23). These approaches have demonstrated that neuronal activity directly influences intracellular redox homeostasis through dynamic regulation of mitochondrial respiration, calcium signaling, and metabolic demand.

Recent advances in photo-oxidative neuromodulation have further expanded the application of optogenetics to redox biology. Selective optical manipulation of neuronal firing patterns has revealed that physiological neuronal activity promotes activation of endogenous antioxidant signaling pathways, particularly the nuclear factor erythroid 2-related factor 2 (Nrf2) pathway, which regulates transcription of numerous cytoprotective genes including heme oxygenase-1 (HO-1), NAD(P)H quinone oxidoreductase-1 (NQO1), glutamate-cysteine ligase (GCL), and superoxide dismutase (SOD). Conversely, prolonged hyperexcitability markedly increases mitochondrial ROS generation, suppresses antioxidant defenses, and sensitizes neurons to oxidative injury (80). These findings indicate that neuronal activity exerts bidirectional effects on redox homeostasis depending upon its intensity and duration.

Optogenetic investigations have also highlighted the close interplay between oxidative stress, neuroinflammation, and mitochondrial dysfunction. Excessive ROS generated during neuronal hyperactivity activate microglia and astrocytes through redox-sensitive transcription factors such as NF-*κ*B, leading to sustained production of inflammatory cytokines including tumor necrosis factor-α (TNF-α), interleukin-1β (IL-1β), and interleukin-6 (IL-6). These inflammatory mediators further enhance oxidative damage by stimulating NOX activity and impairing mitochondrial function, thereby establishing a vicious cycle that progressively exacerbates neuronal injury (71). Optogenetic modulation of cortical and hippocampal circuits has demonstrated that restoration of physiological neuronal activity attenuates oxidative stress while simultaneously reducing neuroinflammatory responses, emphasizing the intimate relationship between neuronal excitability and cellular redox balance.

Another important contribution of optogenetics has been the investigation of oxidative stress during synaptic dysfunction. Reactive oxygen species profoundly influence neurotransmitter release, receptor trafficking, dendritic spine morphology, and synaptic plasticity. Moderate ROS concentrations participate in long-term potentiation (LTP) and memory formation, whereas excessive ROS disrupt glutamatergic transmission, inhibit N-methyl-D-aspartate (NMDA) receptor signaling, and facilitate long-term depression (LTD). Using pathway-specific optical stimulation, investigators have demonstrated that oxidative stress selectively impairs hippocampal and cortical synaptic plasticity before overt neuronal degeneration becomes apparent, suggesting that redox imbalance represents one of the earliest functional abnormalities during neurodegenerative disease progression (16).

Emerging optogenetic technologies are increasingly integrated with advanced metabolomics, redox imaging, mitochondrial biosensors, and artificial intelligence-assisted image analysis to characterize oxidative dynamics at single-cell resolution. Such multidisciplinary approaches permit simultaneous monitoring of neuronal activity, mitochondrial metabolism, calcium signaling, and ROS production within intact neural circuits, thereby providing comprehensive insight into the molecular events linking neuronal activity to oxidative injury. Furthermore, development of photoactivatable antioxidant systems and optogenetic regulation of intracellular signaling pathways may offer novel opportunities to selectively enhance endogenous neuroprotective mechanisms while minimizing systemic side effects.

### 5.6 Cell death pathways

Progressive neuronal loss is the pathological hallmark of neurodegenerative disorders and results from activation of multiple regulated cell death pathways rather than passive degeneration. Recent evidence indicates that apoptosis, ferroptosis, pyroptosis, and necroptosis interact closely with mitochondrial dysfunction, oxidative stress, neuroinflammation, and protein aggregation to drive neuronal death. Optogenetics has become an invaluable tool for dissecting these mechanisms by enabling precise modulation of neuronal activity and monitoring downstream molecular responses within disease-relevant neural circuits. Unlike conventional biochemical approaches, optogenetics establishes causal relationships between neuronal excitability and activation of specific cell death pathways, thereby providing important mechanistic insights into disease progression (10, 16).

#### 5.6.1 Apoptosis

Apoptosis is the best-characterized programmed cell death pathway involved in neurodegeneration and is primarily mediated through intrinsic mitochondrial and extrinsic death receptor pathways. Cellular stress caused by oxidative damage, DNA injury, mitochondrial dysfunction, calcium overload, and protein aggregation activates pro-apoptotic proteins such as Bax and Bak, resulting in mitochondrial outer membrane permeabilization and release of cytochrome c. Subsequent activation of caspase-9 and caspase-3 initiates irreversible degradation of intracellular proteins, DNA fragmentation, and neuronal death (81).

Optogenetic investigations have demonstrated that abnormal neuronal activity directly influences apoptotic signaling. Sustained neuronal hyperexcitability increases intracellular calcium influx and mitochondrial depolarization, thereby activating Bax-dependent apoptosis. Conversely, restoration of physiological neuronal firing through selective optogenetic neuromodulation suppresses caspase activation, preserves mitochondrial integrity, and improves neuronal survival in experimental models of Alzheimer's disease, Parkinson's disease, and cerebral ischemia (16). These findings indicate that apoptosis is closely linked to neural circuit dysfunction and may be partially reversible during early stages of neurodegeneration.

#### 5.6.2 Ferroptosis

Ferroptosis is a recently recognized iron-dependent form of regulated cell death characterized by excessive lipid peroxidation and catastrophic membrane damage. Unlike apoptosis, ferroptosis is independent of caspase activation and is initiated by intracellular iron accumulation, glutathione depletion, impaired glutathione peroxidase-4 (GPX4) activity, and overwhelming oxidative stress. Because the brain contains abundant iron and highly oxidizable membrane lipids, neurons are particularly susceptible to ferroptotic injury (82). Growing evidence implicates ferroptosis in Alzheimer's disease, Parkinson's disease, Huntington's disease, and amyotrophic lateral sclerosis. Optogenetic modulation of neuronal activity has revealed that excessive neuronal firing increases mitochondrial ROS production and lipid oxidation, thereby sensitizing neurons to ferroptotic death. Conversely, normalization of neuronal activity reduces oxidative stress, preserves GPX4 activity, and limits lipid peroxidation. Integration of optogenetics with genetically encoded redox sensors has enabled real-time visualization of oxidative events preceding ferroptosis, providing valuable insights into how neuronal metabolism and circuit activity regulate iron-dependent neurodegeneration (23).

#### 5.6.3 Pyroptosis

Pyroptosis is a highly inflammatory form of programmed cell death mediated by inflammasome activation and gasdermin-dependent membrane pore formation. Activation of the NLRP3 inflammasome promotes cleavage of pro-caspase-1, resulting in maturation of interleukin-1β (IL-1β) and interleukin-18 (IL-18), cleavage of gasdermin D, membrane rupture, and release of pro-inflammatory mediators. Persistent inflammasome activation contributes significantly to chronic neuroinflammation and progressive neuronal damage in Alzheimer's disease and Parkinson's disease (71, 83). Optogenetic studies have revealed that neuronal activity influences inflammasome signaling through regulation of mitochondrial ROS production, ATP release, calcium homeostasis, and neuron–microglia communication. Gamma-frequency optogenetic stimulation has been shown to reduce inflammatory activation, suppress NLRP3 signaling, enhance microglial phagocytosis, and decrease amyloid pathology in Alzheimer's disease models (27). These observations demonstrate that modulation of neuronal circuits can indirectly regulate innate immune responses and attenuate pyroptotic cell death.

#### 5.6.4 Necroptosis

Necroptosis is a caspase-independent regulated necrotic pathway mediated by receptor-interacting protein kinases RIPK1 and RIPK3 together with mixed lineage kinase domain-like protein (MLKL). Following activation, phosphorylated MLKL oligomerizes within the plasma membrane, causing membrane rupture, cellular swelling, and release of intracellular damage-associated molecular patterns (DAMPs), thereby amplifying neuroinflammatory responses (84). Increasing evidence suggests that necroptosis contributes to neuronal degeneration following ischemic stroke, traumatic brain injury, multiple sclerosis and several chronic neurodegenerative disorders. Optogenetic manipulation has demonstrated that prolonged neuronal hyperactivity promotes activation of RIPK signaling pathways through mitochondrial dysfunction, calcium overload, and oxidative stress, whereas restoration of physiological circuit activity reduces necroptotic signaling and preserves neuronal viability. These findings further support the concept that regulated necrosis is closely linked to neural circuit dysfunction and may represent a promising therapeutic target.

### 5.7 Evidence maturity and translational status of optogenetic approaches

Optogenetics has emerged as a powerful experimental platform for establishing causal relationships between neuronal activity, circuit dysfunction, and behavioural outcomes; however, its current applications in neurodegenerative disorders should primarily be considered as mechanistic research tools rather than clinically validated therapeutic approaches (16, 23, 25). Most studies in AD, PD, HD, ALS, MS and stroke have been performed in genetically modified rodent models, where cell-type-specific opsin expression and precise optical stimulation enable detailed investigation of disease-associated neural circuits (10, 16, 25). These studies have provided important insights into memory engrams, basal ganglia dysfunction, synaptic plasticity, neuroimmune regulation and activity-dependent circuit recovery (16, 26–28). However, functional improvements observed in preclinical models cannot be directly interpreted as clinical efficacy because human neurodegenerative disorders involve complex genetic backgrounds, heterogeneous progression patterns, extensive neuronal loss and multifactorial pathological mechanisms that are not completely reproduced in experimental models (2, 3, 25).

In AD models, optogenetic activation of hippocampal engram cells and gamma-frequency stimulation have improved memory-associated behaviours and modulated amyloid-related processes; however, their durability, safety and therapeutic relevance in humans remain uncertain (26, 27). Similarly, although optogenetic manipulation of dopaminergic and basal ganglia circuits has advanced understanding of PD pathophysiology and validated circuit-based therapeutic concepts, clinical translation requires overcoming challenges related to targeted gene delivery, long-term opsin expression, immune compatibility and deep-brain optical access (23, 25, 28). Comparable limitations exist across other neurodegenerative disorders, where optogenetics has primarily contributed to mechanistic understanding and target identification rather than demonstrated therapeutic efficacy in patients (10, 16). Therefore, current evidence supports optogenetics as a transformative neuroscience tool for dissecting disease mechanisms and evaluating circuit-based interventions, whereas its role as a clinical neuromodulation strategy remains investigational. Future translation will require large-animal validation, long-term biosafety assessment, optimized delivery systems, clinically compatible optical interfaces, regulatory evaluation and carefully designed human studies to determine whether experimental circuit modulation can be converted into safe and effective therapies (7, 23, 25).

## 6 Optogenetic applications in neurodegenerative disorders

Neurodegenerative disorders, including AD, PD, HD, ALS, MS and stroke-related neurodegeneration, are characterized by progressive neuronal loss, synaptic dysfunction, impaired neural connectivity, and chronic neuroinflammation. Although current therapies primarily provide symptomatic relief, they fail to halt disease progression or restore damaged neural circuits. Optogenetics has emerged as a transformative approach for investigating these disorders by enabling cell type-specific activation or inhibition of genetically defined neuronal populations with millisecond temporal precision and high spatial resolution. Unlike pharmacological or electrical stimulation, optogenetics permits precise dissection of disease-associated neural circuits, revealing that early abnormalities in network activity, synaptic plasticity, and excitatory–inhibitory balance often precede overt neurodegeneration (15, 16). Experimental studies have demonstrated that optogenetic modulation of hippocampal, basal ganglia, corticostriatal, corticospinal, and sensorimotor circuits can restore physiological neuronal activity, improve cognitive and motor functions, enhance neuroplasticity, promote remyelination, and facilitate functional recovery. Furthermore, integration of optogenetics with advanced imaging, electrophysiology, artificial intelligence, and regenerative therapies is accelerating the development of circuit-based precision neurotherapeutics. The following sections summarize recent advances in applying optogenetics to major neurodegenerative disorders and highlight its potential for both mechanistic investigations and targeted therapeutic interventions.

### 6.1 Alzheimer's disease

Optogenetics has emerged as a transformative approach for elucidating the circuit-level mechanisms underlying AD by enabling precise temporal and spatial control of genetically defined neuronal populations. Unlike pharmacological or electrical stimulation, optogenetics allows selective modulation of specific neuronal circuits, facilitating detailed investigation of how network dysfunction contributes to cognitive decline, synaptic failure, and disease progression. Studies in transgenic AD models have demonstrated that optogenetic manipulation can restore impaired neuronal communication, improve cognitive performance, and clarify the relationship between neuronal activity and pathological protein accumulation (10, 16).

#### 6.1.1 Hippocampal circuits

The hippocampus is among the earliest brain regions affected in AD, exhibiting synaptic loss, impaired long-term potentiation (LTP), neuronal degeneration, and disrupted information processing. Optogenetic activation of excitatory neurons within the dentate gyrus (DG), CA3, and CA1 subfields has demonstrated that cognitive deficits frequently result from dysfunctional circuit activity rather than irreversible neuronal loss. Reactivation of hippocampal memory engram cells restores contextual and spatial memory retrieval in AD mouse models, indicating that memory traces remain stored but become inaccessible because of impaired network connectivity (26). Furthermore, optogenetic regulation of parvalbumin-positive and somatostatin-positive interneurons has revealed that restoration of the excitatory–inhibitory balance improves synaptic plasticity, neuronal synchronization, and memory encoding (10, 85). These findings identify hippocampal circuitry as a major therapeutic target for reversing cognitive dysfunction.

#### 6.1.2 Gamma oscillations

Gamma oscillations (30–80 Hz) are essential for neuronal synchrony underlying learning, attention, and memory but are markedly disrupted in AD. Optogenetic stimulation of parvalbumin-positive interneurons at approximately 40 Hz entrains gamma oscillations, restores hippocampal-cortical synchrony, and improves cognitive performance. Importantly, 40-Hz gamma entrainment reduces amyloid-β deposition by promoting microglial activation and enhancing phagocytic clearance of amyloid plaques. Gamma stimulation also suppresses neuroinflammation, preserves synaptic integrity, and improves neuronal network function (27, 86). These findings demonstrate that gamma oscillations regulate both neuronal communication and immune-mediated clearance of pathological proteins, highlighting neuronal rhythm modulation as a promising therapeutic strategy.

#### 6.1.3 Amyloid and tau pathology

Optogenetics has substantially improved understanding of how neuronal activity regulates amyloid-β and tau pathology. Experimental manipulation of neuronal firing demonstrates that sustained neuronal hyperactivity accelerates amyloid precursor protein processing, increasing amyloid-β production and extracellular plaque formation. Likewise, aberrant neuronal activity facilitates trans-synaptic propagation of hyperphosphorylated tau, accelerating disease spread across anatomically connected neural networks (87, 88). Conversely, normalization of network activity through optogenetic modulation decreases pathological protein accumulation, attenuates synaptic dysfunction, and limits neurodegeneration. These studies support the concept that AD progression is strongly influenced by activity-dependent mechanisms and that modulation of dysfunctional circuits may slow disease progression alongside reducing pathological protein burden.

#### 6.1.4 Memory restoration

One of the most compelling findings from optogenetic studies is that memory deficits in AD may reflect impaired retrieval rather than complete erasure of stored information. Selective optical reactivation of hippocampal memory engram cells successfully restores contextual and spatial memories in transgenic AD mice despite persistent amyloid pathology (26). Repeated optogenetic stimulation further enhances LTP, strengthens hippocampal-prefrontal cortical connectivity, improves synaptic efficacy, and enhances cognitive performance (85). These observations suggest that restoring circuit function may complement disease-modifying therapies by recovering cognitive abilities even in the presence of ongoing neuropathology. Collectively, optogenetic investigations have established AD as a disorder of dysfunctional neural networks and demonstrate that precise circuit modulation can simultaneously improve memory, restore synaptic function, and reduce pathological processes, supporting the development of next-generation circuit-based therapeutic interventions.

### 6.2 Parkinson's disease

Parkinson's disease (PD) is a progressive neurodegenerative disorder characterized by the degeneration of dopaminergic neurons in the substantia nigra pars compacta (SNpc), resulting in dopamine depletion within the striatum and disruption of basal ganglia circuitry. Clinically, PD manifests as bradykinesia, resting tremor, rigidity, postural instability, and progressive cognitive impairment. Although pharmacological therapies and deep-brain stimulation (DBS) provide symptomatic relief, they do not fully restore physiological circuit function or prevent disease progression. Optogenetics has transformed PD research by enabling cell-type-specific manipulation of neuronal populations, allowing precise dissection of motor circuit dysfunction and identification of therapeutic targets beyond conventional electrical stimulation (16, 89).

#### 6.2.1 Basal ganglia circuitry

The basal ganglia regulate voluntary movement through the coordinated activity of the direct and indirect pathways. Dopamine depletion disrupts this balance by reducing activity in dopamine D1 receptor-expressing direct pathway spiny projection neurons (dSPNs) while enhancing activity in dopamine D2 receptor-expressing indirect pathway neurons (iSPNs), leading to excessive inhibitory output from the globus pallidus interna and substantia nigra pars reticulata. Optogenetic studies have demonstrated that selective activation of dSPNs rapidly improves locomotion, whereas stimulation of iSPNs induces Parkinsonian motor deficits in healthy animals. Conversely, inhibition of the indirect pathway alleviates akinesia and rigidity, confirming that pathway-specific imbalance underlies PD motor symptoms (50, 89). These findings have fundamentally reshaped the understanding of basal ganglia physiology and established circuit-specific neuromodulation as a promising therapeutic strategy.

#### 6.2.2 Dopaminergic neurons

Selective optogenetic manipulation of dopaminergic neurons in the SNpc and ventral tegmental area has provided direct evidence for the causal relationship between dopamine signaling and motor control. Optical stimulation of surviving dopaminergic neurons enhances dopamine release in the striatum, improves motor coordination, and partially restores normal movement in experimental PD models (90). Beyond motor regulation, optogenetics has revealed the functional heterogeneity of dopaminergic neurons, demonstrating that distinct neuronal populations differentially regulate movement initiation, reward processing, motivation, and motor learning. Moreover, optogenetic approaches have been widely used to evaluate stem cell-derived dopaminergic neuron transplantation by assessing functional integration of grafted neurons into host motor circuits, thereby facilitating the development of regenerative therapies (28).

#### 6.2.3 Motor recovery

Optogenetic modulation has shown significant potential for restoring motor function by re-establishing physiological neuronal activity within impaired motor circuits. Stimulation of motor cortical projections, corticostriatal pathways, and surviving nigrostriatal dopaminergic neurons enhances locomotor performance, improves gait, and reduces bradykinesia in animal models of PD. In addition to immediate motor improvement, repeated optogenetic stimulation promotes activity-dependent synaptic plasticity, strengthens corticostriatal connectivity, and facilitates functional reorganization of motor networks (89). These findings suggest that circuit-specific neuromodulation may produce sustained therapeutic benefits by enhancing adaptive neuroplasticity rather than merely compensating for dopamine deficiency.

#### 6.2.4 Deep-brain stimulation optimization

Deep-brain stimulation of the subthalamic nucleus (STN) and globus pallidus interna remains an established treatment for advanced PD; however, its mechanisms of action have remained incompletely understood because electrical stimulation activates both neuronal cell bodies and passing axons indiscriminately. Optogenetics has helped clarify these mechanisms by selectively stimulating or inhibiting individual neuronal populations within DBS-targeted circuits. Experimental studies indicate that therapeutic benefits primarily arise from modulation of specific afferent cortical axons and downstream basal ganglia networks rather than simple suppression of STN neuronal activity (28). These discoveries have informed the refinement of DBS parameters, improved understanding of stimulation-induced network dynamics, and supported the development of next-generation closed-loop neuromodulation systems capable of delivering adaptive, circuit-specific stimulation with reduced adverse effects. Collectively, optogenetic investigations have substantially advanced knowledge of PD pathophysiology by linking basal ganglia circuit dysfunction with motor impairment and identifying precise neuronal targets for more effective neuromodulatory therapies.

### 6.3 Huntington's disease

Huntington's disease (HD) is an autosomal dominant neurodegenerative disorder caused by expanded CAG repeats in the *HTT* gene, leading to the production of mutant huntingtin (mHTT) protein. Progressive degeneration of *γ*-aminobutyric acid (GABA)-ergic medium spiny neurons (MSNs) within the striatum disrupts corticostriatal communication, resulting in chorea, dystonia, cognitive decline, and psychiatric disturbances. Optogenetics has become an indispensable tool for elucidating HD pathophysiology by enabling selective modulation of defined neuronal populations and revealing how dysfunction within corticostriatal circuits contributes to motor and cognitive impairment (16, 91).

#### 6.3.1 Corticostriatal pathways

The corticostriatal pathway, which connects glutamatergic neurons of the motor cortex with striatal MSNs, plays a central role in voluntary movement and motor learning. In HD, mutant huntingtin disrupts excitatory synaptic transmission, impairs long-term potentiation (LTP), enhances long-term depression (LTD), and alters the balance between the direct and indirect basal ganglia pathways. Optogenetic stimulation of cortical afferents has demonstrated that early synaptic dysfunction precedes overt neuronal degeneration, indicating that circuit abnormalities are among the earliest pathological events in HD (91). Furthermore, pathway-specific activation has shown that selective restoration of corticostriatal activity improves synaptic transmission, neuronal excitability, and activity-dependent plasticity. Optogenetic interrogation has also identified abnormalities in cortical interneurons and thalamostriatal projections, revealing that HD involves widespread network dysfunction extending beyond the striatum. These findings provide important mechanistic insights into disease progression and identify corticostriatal connectivity as a promising therapeutic target.

#### 6.3.2 Motor dysfunction

Motor impairment in HD results from progressive disruption of basal ganglia output caused by degeneration of striatal MSNs and impaired communication between cortical and subcortical motor networks. Optogenetic studies have demonstrated that selective activation of surviving corticostriatal projections or direct pathway neurons improves locomotor activity, movement initiation, and motor coordination in transgenic HD models, whereas inhibition of hyperactive indirect pathway circuits reduces abnormal involuntary movements (89). Repeated optogenetic stimulation further enhances synaptic plasticity, promotes functional reorganization of motor circuits, and partially restores balanced neuronal activity within the basal ganglia. Beyond motor control, optogenetic investigations have revealed that dysfunction of corticostriatal networks also contributes to impaired procedural learning, executive dysfunction, and behavioral abnormalities observed in HD. Collectively, these findings demonstrate that circuit dysfunction precedes extensive neuronal loss and suggest that early circuit-specific neuromodulation may delay functional decline. Consequently, optogenetics provides a valuable experimental platform for evaluating disease-modifying interventions, including gene-editing strategies, RNA-targeted therapies, neuroprotective agents, and cell replacement approaches aimed at preserving corticostriatal integrity and improving motor function.

### 6.4 Amyotrophic lateral sclerosis

Amyotrophic lateral sclerosis (ALS) is a fatal neurodegenerative disorder characterized by the progressive degeneration of upper and lower motor neurons, resulting in muscle weakness, spasticity, paralysis, and ultimately respiratory failure. Although the precise mechanisms underlying ALS remain incompletely understood, accumulating evidence indicates that neuronal hyperexcitability, glutamate excitotoxicity, mitochondrial dysfunction, neuroinflammation, and impaired synaptic connectivity contribute to disease initiation and progression. Optogenetics has provided unprecedented opportunities to investigate these mechanisms by enabling selective manipulation of motor neurons and associated neural circuits with high temporal precision, thereby facilitating the identification of early pathological changes and potential therapeutic targets (15, 16).

#### 6.4.1 Motor neuron degeneration

Degeneration of corticospinal and spinal motor neurons is the pathological hallmark of ALS. Optogenetic approaches have been extensively employed to examine how altered neuronal activity influences motor neuron survival and neuromuscular function. Selective optical stimulation of corticospinal neurons and spinal motor neurons has demonstrated that excessive neuronal firing accelerates excitotoxic injury, whereas controlled modulation of neuronal activity improves synaptic stability and preserves neuromuscular junction integrity in experimental models (92). Optogenetics has also enabled precise functional assessment of surviving motor neurons during disease progression, revealing that synaptic dysfunction and impaired motor circuit connectivity precede overt neuronal loss. Furthermore, combining optogenetics with calcium imaging and electrophysiological recordings has clarified how abnormalities in intracellular calcium homeostasis contribute to progressive motor neuron degeneration. These findings indicate that activity-dependent mechanisms play a pivotal role in ALS pathogenesis and highlight neuronal excitability as a promising therapeutic target.

#### 6.4.2 Cortical hyperexcitability

Cortical hyperexcitability is increasingly recognized as an early biomarker of ALS, often preceding clinical symptoms and widespread neurodegeneration. Increased excitability of motor cortical neurons enhances glutamatergic transmission, promoting excitotoxic damage to spinal motor neurons and accelerating disease progression. Optogenetic studies have demonstrated that selective modulation of excitatory pyramidal neurons and inhibitory interneurons restores the excitatory–inhibitory balance within the motor cortex, thereby reducing pathological network activity (15). Experimental manipulation of corticospinal circuits has further shown that normalization of cortical activity improves motor output, preserves downstream spinal connectivity, and delays functional deterioration in preclinical models. In addition, optogenetic interrogation of astrocyte–neuron and microglia–neuron interactions has revealed that non-neuronal cells significantly influence cortical excitability through the release of inflammatory mediators and altered glutamate homeostasis. These observations emphasize that ALS is a network disorder involving reciprocal dysfunction among neurons and glial cells rather than isolated motor neuron degeneration. Consequently, optogenetics serves as a powerful platform for evaluating circuit-based interventions, gene therapies, and neuroprotective strategies aimed at reducing hyperexcitability, preserving motor neuron function, and slowing disease progression.

### 6.5 Multiple sclerosis

Multiple sclerosis (MS) is a chronic autoimmune demyelinating disorder of the central nervous system characterized by inflammatory demyelination, axonal degeneration, gliosis, and progressive neurological disability. Loss of myelin disrupts saltatory conduction, impairs neuronal communication, and ultimately leads to motor, sensory, visual, and cognitive dysfunction. Although current immunomodulatory therapies reduce inflammatory relapses, they have limited efficacy in promoting remyelination and restoring neural circuit function. Optogenetics has emerged as a valuable experimental approach for investigating activity-dependent mechanisms that regulate myelin repair, oligodendrocyte function, and functional recovery by enabling precise control of neuronal activity within affected circuits (15, 16).

#### 6.5.1 Demyelination

Optogenetic studies have demonstrated that neuronal activity plays a fundamental role in maintaining myelin integrity and regulating neuron–glia communication. In experimental demyelination models, selective stimulation of cortical and corticospinal neurons enhances activity-dependent signaling to oligodendrocyte precursor cells (OPCs), while altered neuronal firing contributes to axonal dysfunction and impaired signal transmission. Optical manipulation of specific neural circuits has shown that reduced neuronal activity exacerbates conduction deficits and synaptic dysfunction, whereas physiological stimulation partially restores neuronal connectivity by improving axonal conduction and synaptic transmission (60). Furthermore, optogenetic approaches have enabled detailed investigation of interactions between neurons, oligodendrocytes, astrocytes, and microglia, revealing that inflammatory mediators and disrupted electrical activity jointly influence the progression of demyelination. These findings demonstrate that neuronal activity is an important regulator of both disease progression and circuit dysfunction in MS.

#### 6.5.2 Remyelination

Remyelination is a critical regenerative process that restores axonal conduction and protects neurons from irreversible degeneration. Optogenetic studies have shown that activity-dependent stimulation promotes OPC proliferation, migration, differentiation into mature oligodendrocytes, and subsequent myelin formation around damaged axons. Enhanced neuronal firing increases the release of neurotransmitters and trophic factors that stimulate myelin repair and improve structural and functional recovery (93). In addition, optogenetic activation of motor and sensory pathways accelerates restoration of conduction velocity, strengthens synaptic plasticity, and improves behavioral performance in experimental models of demyelination. These observations indicate that neural activity serves as a physiological signal coordinating communication between neurons and oligodendrocytes during myelin regeneration. Consequently, optogenetics provides a powerful platform for evaluating remyelinating therapies, stem cell-based interventions, biomaterial-assisted regeneration, and neuromodulatory strategies aimed at enhancing endogenous repair mechanisms (94). Collectively, these studies establish that neuronal activity not only regulates myelin maintenance but also facilitates functional recovery following demyelinating injury, supporting the development of activity-dependent therapeutic approaches for progressive MS.

### 6.6 Stroke and brain injury

Stroke and traumatic brain injury (TBI) are major causes of long-term neurological disability, resulting from acute neuronal loss, disruption of neural circuits, neuroinflammation, and impaired synaptic connectivity. Although spontaneous recovery occurs to some extent through neuroplasticity, functional restoration is often incomplete because of limited regeneration of damaged neural networks. Optogenetics has become an important experimental tool for investigating post-injury circuit reorganization by enabling precise modulation of defined neuronal populations with high temporal and spatial resolution. This approach has significantly improved understanding of the mechanisms underlying cortical plasticity and has identified novel strategies for enhancing functional recovery after brain injury (15, 16).

#### 6.6.1 Cortical plasticity

Recovery following stroke or TBI depends largely on the capacity of surviving neuronal networks to undergo structural and functional reorganization. Optogenetic stimulation of neurons within the peri-infarct cortex, motor cortex, and corticospinal pathways has demonstrated that activity-dependent neuronal activation enhances synaptic plasticity, promotes dendritic spine remodeling, strengthens long-term potentiation (LTP), and facilitates the formation of new functional connections (95). Furthermore, selective modulation of excitatory pyramidal neurons and inhibitory interneurons restores the excitatory–inhibitory balance disrupted after injury, thereby improving cortical network synchronization and information processing. Optogenetic studies have also shown that coordinated activation of corticocortical and corticothalamic circuits enhances axonal sprouting and compensatory network reorganization, indicating that targeted circuit modulation can accelerate endogenous neuroplastic mechanisms. These findings establish cortical plasticity as a key determinant of neurological recovery and identify neuronal activity as a major driver of post-injury circuit remodeling.

#### 6.6.2 Functional recovery

Beyond elucidating mechanisms of neural repair, optogenetics has demonstrated considerable potential for promoting functional recovery after stroke and TBI. Optical stimulation of motor cortical neurons, corticospinal projections, and sensorimotor pathways improves motor coordination, gait, forelimb function, and skilled motor performance in experimental models. Repeated stimulation further enhances activity-dependent neurogenesis, angiogenesis, synaptogenesis, and axonal regeneration, contributing to sustained improvements in neurological function (96, 97). In addition, optogenetic modulation reduces maladaptive network hyperexcitability, limits secondary neuronal damage, and improves functional connectivity between ipsilateral and contralateral cortical regions. When combined with stem cell transplantation, biomaterial scaffolds, rehabilitation, or pharmacological interventions, optogenetics provides a powerful platform for evaluating synergistic regenerative strategies and optimizing therapeutic timing. Collectively, these studies demonstrate that targeted modulation of neural circuits can enhance neuroplasticity, accelerate functional recovery, and improve behavioral outcomes following cerebral ischemia or traumatic brain injury. Although current applications remain largely preclinical, advances in optogenetic technologies and neuromodulation continue to provide valuable insights for developing next-generation precision therapies aimed at restoring neural circuit function after neurological injury.

### 6.7 Interpretation of therapeutic outcomes across neurodegenerative diseases

Reported benefits of optogenetic manipulation should be interpreted according to the outcome measured and its relationship to underlying disease pathology. Symptomatic circuit modulation refers to improvements in motor, cognitive, or other functional endpoints during or shortly after stimulation without demonstrating altered disease progression. Functional compensation denotes recruitment or reorganization of surviving circuits that improves performance despite persisting pathology. Neuroprotection requires evidence that an intervention preserves vulnerable neurons, axons, synapses, or myelin relative to appropriate disease controls. Disease modification demands a stronger longitudinal demonstration that the intervention alters the trajectory of the pathological process or sustained clinical-relevant decline, rather than only producing transient functional improvement. These categories are not interchangeable, and a positive behavioural endpoint alone cannot establish neuroprotection or disease modification (10, 16, 23).

Across disease models, reactivation of memory engram cells in Alzheimer's disease supports functional retrieval despite persistent amyloid pathology (26), whereas gamma-frequency entrainment has been associated with changes in amyloid-related and microglial endpoints in particular experimental models (27, 86). In Parkinson's disease, pathway-specific basal ganglia stimulation primarily demonstrates acute motor circuit modulation (28, 50); graft interrogation establishes functional integration rather than durable disease reversal (115). Evidence discussed for Huntington's disease and amyotrophic lateral sclerosis predominantly informs circuit dysfunction or excitability and does not independently establish modification of disease progression (91, 92). Activity-dependent oligodendrogenesis provides mechanistic support for adaptive myelination, but this should not be equated with clinical remyelination in multiple sclerosis (93). Likewise, improvements after experimental post-stroke stimulation require separation from spontaneous recovery and general rehabilitation effects. Future studies should include stimulation-off follow-up, blinded functional endpoints, neuropathological measures, appropriate disease controls, and longitudinal assessment to determine the durability and biological meaning of reported improvements.

## 7 Emerging therapeutic strategies

Recent advances in molecular engineering, neurotechnology and computational neuroscience have expanded the experimental capabilities of optogenetics beyond conventional circuit manipulation. Integration with closed-loop control systems, wireless photonic devices, artificial intelligence, brain computer interfaces, nanotechnology and regenerative approaches has introduced new strategies for improving spatiotemporal precision and understanding complex neural circuit dynamics. However, the translational maturity of these emerging technologies varies considerably, with most approaches remaining at experimental or preclinical stages. Although these platforms provide promising opportunities for future precision neuromodulation, their clinical implementation requires further validation regarding long-term safety, biological compatibility, delivery efficiency and regulatory feasibility. The current evidence status, demonstrated applications, and major translational challenges associated with emerging optogenetic technologies are summarized in Table 2. The emerging therapeutic platforms integrating optogenetics with advanced neurotechnologies are illustrated in Figure 4, while the following sections discuss recent developments in closed-loop optogenetics, wireless neuromodulation, artificial intelligence-assisted optogenetics, brain computer interfaces, nanotechnology-enabled light delivery and combination approaches involving stem cells and gene editing.

**Table 2:** Current evidence status and translational challenges of emerging optogenetic technologies.

Technology	Demonstrated achievements	Current evidence level	Major translational challenges	Reference
Closed-loop optogenetics	Real-time neural recording with adaptive stimulation in experimental models of epilepsy, Parkinson's disease, and movement disorders	Preclinical	Reliable biomarkers, long-term stability, hardware integration, clinical validation	(98, 101)
Wireless neuromodulation	Untethered optical stimulation using miniaturized implants in freely moving animal models	Preclinical	Power supply, thermal effects, implant biocompatibility, long-term safety	(117)
AI-assisted optogenetics	Analysis of large-scale neural datasets and optimization of stimulation parameters	Experimental/ preclinical	Dataset quality, interpretability, biological validation, regulatory acceptance	(101, 103, 104)
Brain-computer interfaces	Bidirectional communication between neural signals and external devices	Developing technology	Signal stability, invasive procedures, ethical considerations	(106–108)
Nanotechnology-enabled light delivery	Improved optical conversion and potential deep-tissue stimulation approaches	Proof-of-concept/ preclinical	Biodistribution, toxicity, material safety, regulatory approval	(109, 116)
Stem cell and gene-editing integration	Investigation of neural repair, regeneration, and circuit modification strategies	Early experimental stage	Safety, immune response, genomic stability, clinical feasibility	(111, 112, 115)

**Figure 4:**
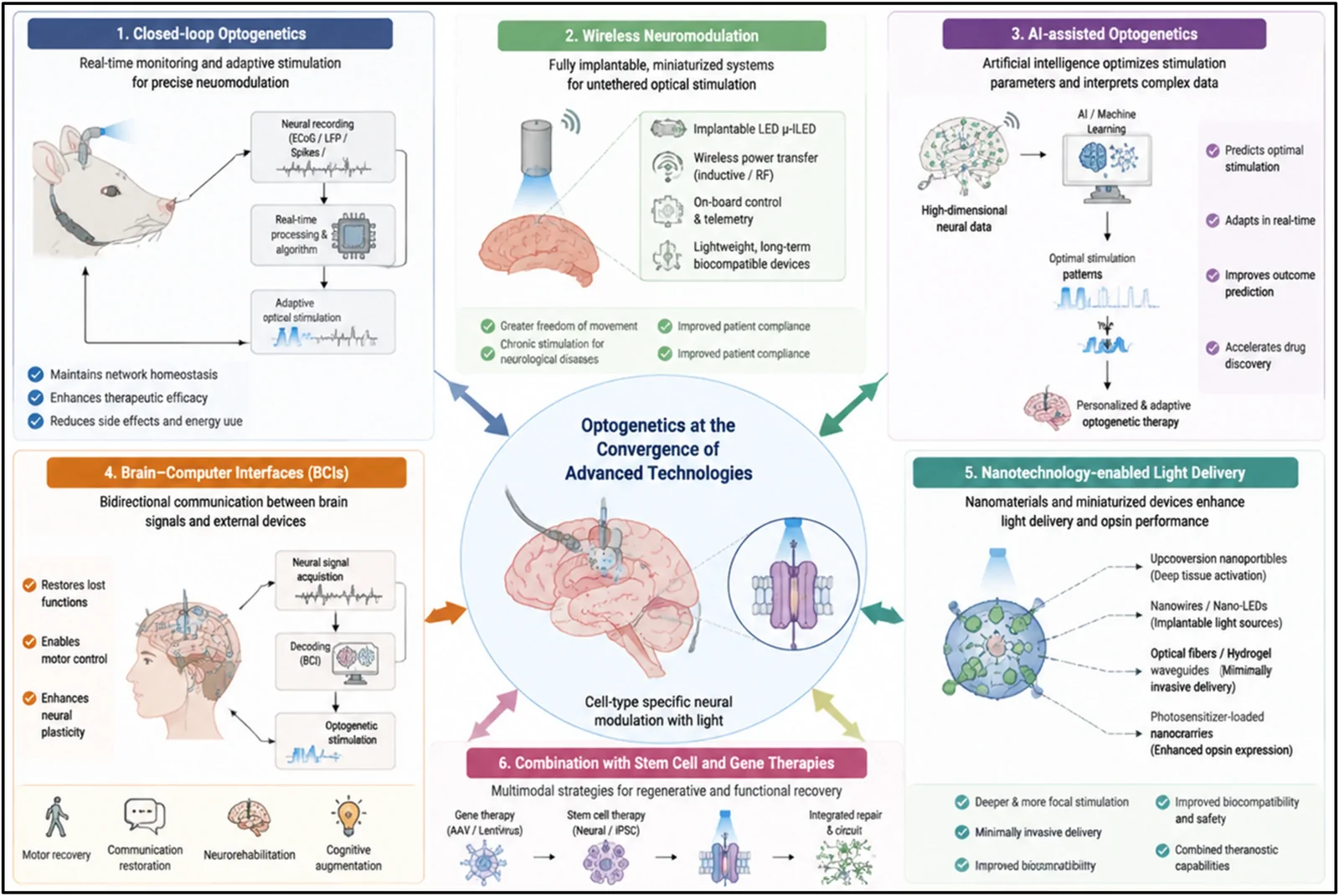
Emerging therapeutic platforms integrating optogenetics with advanced neurotechnologies. Closed loop optogenetics enables real time monitoring and adaptive optical stimulation through feedback-controlled neuromodulation (1). Wireless neuromodulation employs implantable miniaturized photonic devices for untethered stimulation (2), while artificial intelligence optimizes stimulation parameters and therapeutic outcomes using data driven algorithms (3). Brain computer interfaces facilitate bidirectional communication between neural circuits and external devices for functional restoration (4). Nanotechnology enhances light delivery and opsin performance using nanoparticles, nanowires, and minimally invasive optical systems (5). Integration of optogenetics with stem cell and gene therapies promotes neural regeneration and circuit repair (6). Collectively, these converging technologies expand the experimental capabilities of optogenetics and may contribute to future precision neuromodulation strategies; however, clinical translation requires further biological, engineering and regulatory validation.

### 7.1 Closed-loop optogenetics

Conventional optogenetic stimulation generally operates in an open-loop manner, delivering predefined light patterns irrespective of ongoing neuronal activity. In contrast, closed-loop optogenetics combines real-time neural recording with automated optical stimulation, allowing continuous monitoring of brain activity and immediate adjustment of stimulation parameters. Electrophysiological recordings, calcium imaging or electroencephalography (EEG) signals are processed to detect abnormal neuronal activity, which subsequently triggers precisely timed optogenetic intervention. In experimental models of epilepsy, Parkinson's disease and essential tremor, closed-loop approaches have been investigated for detecting and modulating pathological activity. Relative to continuous stimulation, feedback-guided protocols may reduce unnecessary stimulation and energy use, although consistent reductions in adverse effects and improvements in therapeutic outcomes have not been established. Integration with machine learning could support prediction of disease-associated activity and adaptive adjustment of stimulation parameters (16, 98); however, reproducibility, safety and clinical benefits require further validation.

### 7.2 Wireless neuromodulation

One of the principal obstacles to clinical optogenetics is the dependence on tethered optical fibers, which restrict natural movement and increase surgical complexity. Advances in wireless neuromodulation have enabled the development of implantable, miniaturized, battery-free optoelectronic devices capable of delivering light to deep-brain structures while allowing unrestricted behavior. Flexible micro-LEDs, wireless photonic implants and bioresorbable electronic devices offer approaches to spatially controlled stimulation, although tissue responses depend on device design, implementation conditions and duration. Energy harvesting through radiofrequency, near-field communication, ultrasound or photovoltaic systems can reduce reliance on onboard batteries, but sustained operation and chronic implementation remain device-dependent challenges. Wireless systems have demonstrated reliable control of motor behavior, reward circuits and spinal cord activity in freely moving animal models. Their miniaturization, biocompatibility and reduced surgical burden enhance experimental flexibility and may improve future translational feasibility (25, 99–102); however, long-term stability, energy supply, tissue responses and safety remain important challenges.

### 7.3 AI-assisted optogenetics

Artificial intelligence is being explored alongside optogenetics for experimental design, neural decoding and stimulation-parameter selection. Machine learning algorithms can analyze large-scale electrophysiological, calcium imaging and behavioral datasets to identify complex neuronal activity patterns that are difficult to detect using conventional analytical methods (103). AI-assisted systems may enable data-driven adjustment of stimulation frequency, pulse duration, intensity and timing under experimentally validated conditions. Deep-learning approaches may facilitate analysis of complex neural patterns, identification of candidate dysfunctional circuits and optimization of stimulation strategies; however, clinical implementation requires validated datasets, interpretability and robust biological testing. In selected experimental closed-loop platforms, computational algorithms can update stimulation parameters in response to measured neural dynamics, supporting investigation of adaptive neuromodulation approches (104). Integration of AI with optogenetics could improve stimulation precision in experimental settings; however, its application in complex neurological disorders such as epilepsy, Alzheimer's disease and Parkinson's disease requires further validation in diverse biological systems and clinically relevant models (101, 105).

### 7.4 Brain-computer interfaces

Brain-computer interfaces (BCIs) establish communication between neural circuits and external devices and have been investigated for assisting motor, sensory and cognitive functions after neurological injury (106, 107). In experimental settings, combining optogenetics with BCIs offers the possibility of genetically targeted stimulation that conventional electrical interfaces generally cannot provide. Optogenetic BCIs can simultaneously record neuronal activity and deliver targeted optical stimulation, creating bidirectional communication between damaged neural circuits and assistive devices (108). Experimental studies have examined neural decoding sensorimotor responses in models of spinal cord injury and stroke; these findings do not yet establish clinical efficacy of optogenetic BCTs. Integration with robotic prostheses, neuroprosthetic limbs and wearable rehabilitation systems may further enhance functional recovery by promoting activity-dependent cortical plasticity. Further advances in neural recording and adaptive decoding may facilitate evaluation of optogenetic BCIs, but clinical translation will require independent evidence of safety, reliability and functional benefit (15, 16).

### 7.5 Nanotechnology-enabled light delivery

Limited light penetration remains a major barrier to optogenetic therapy, particularly for deep-brain structures. Nanotechnology is being investigated as an alternative means of localized light generation, potentially reducing dependence on implanted optical fibers. Upconversion nanoparticles (UCNPs) convert deeply penetrating near-infrared (NIR) light into visible wavelengths capable of activating microbial opsins (109). Quantum dots, plasmonic nanoparticles and biodegradable nanophotonic materials are also under investigation for localized optical effects, although their photothermal profiles and tissue safety require material-specific assessment (110). Nanomaterials may provide multifunctional experimental platforms that combine imaging, drug delivery and optical stimulation, but proposed theranostic applications remain investigational. Targeted nanoparticle delivery is being studies to improve localization; whether this reduces off-target effects or produces therapeutic benefit in deep-brain disorder remains uncertain. Accordingly, nanotechnology-enabled light delivery should presently be regarded as a preclinical research strategy rather than an established minimally invasive treatment (101, 109).

### 7.6 Combination with stem cell and gene therapies

Combining optogenetics with regenerative medicine represents one of the most promising strategies for restoring neural circuit function following neurodegeneration or injury. Stem cell-derived neurons genetically engineered to express light-sensitive opsins enable precise evaluation of graft survival, neuronal maturation, synaptic integration, and functional connectivity after transplantation (111). Optogenetic stimulation enhances activity-dependent maturation of transplanted neural stem cells, promotes synaptogenesis, and facilitates incorporation into host neural networks. Simultaneously, gene-editing technologies such as CRISPR/Cas systems allow targeted correction of disease-causing mutations while optogenetics provides functional validation of restored neuronal activity (112). Viral vectors including adeno-associated virus (AAV) and lentivirus remain the primary platforms for delivering opsin genes to transplanted or endogenous neurons, although efforts continue to improve targeting specificity and long-term safety (113, 114).

The combination of optogenetics with stem cell therapy has demonstrated encouraging results in experimental models of Parkinson's disease, spinal cord injury, retinal degeneration and stroke, where transplanted neurons establish functional synaptic connections and improve behavioral outcomes. Combining regenerative medicine, gene therapy, biomaterials and adaptive optogenetic neuromodulation may offer routes for investigating circuit repair, but durable functional restoration and personalized clinical treatment have not yet been demonstrated (16, 115).

### 7.7 Evidence maturity and clinical translation boundaries

An important distinction is whether a technology has demonstrated optical control in living animals, therapeutic benefit in a disease model, or reproducible safety and efficacy in humans. These represent separate evidence thresholds. Red-shifted opsins have experimentally useful spectral properties, but longer excitation wavelengths do not eliminate scattering, absorption, dose constraints, or the requirement for spatially controlled illumination (21, 119, 120). Implantable and wireless micro-LED systems enable untethered stimulation in animal models, although chronic thermal, mechanical, power-delivery, and tissue-response constraints remain relevant for human use (117). Closed-loop optogenetic control has been demonstrated experimentally, whereas clinically reliable disease biomarkers, algorithms, and integrated delivery systems are still needed (98). Nanoparticle-mediated near-infrared stimulation has *in vivo* proof-of-concept evidence, but biodistribution, clearance, photothermal dose, repeated administration, and long-term toxicology remain unresolved (109, 116).

Evidence from optogenetic vision-restoration research illustrates that limited human translation is possible in a specific accessible sensory system (48); this should not be extrapolated to deep-brain treatment of neurodegenerative diseases. For intracranial applications, an integrated platform would require reproducible cell-targeted vector delivery, dose-controlled and stable opsin expression, immune monitoring, illumination of anatomically defined targets, chronic device compatibility, demonstrable functional benefit, and an acceptable risk–benefit profile under relevant regulatory oversight. Accordingly, the technologies in Table 2 are classified by their demonstrated experimental status rather than by presumed proximity to clinical approval (23, 113, 117).

## 8 Current challenges and future perspectives

Despite remarkable advances in optogenetics over the past two decades, several technical, biological and translational challenges continue to limit its widespread clinical application. These include inadequate light penetration into deep-brain structures, long-term safety of opsin expression, immune responses to viral vectors and implanted devices and the need for more efficient photosensitive proteins. Advances in molecular engineering, biomaterials, artificial intelligence (AI) and bioelectronics may help address selected limitations, but their contribution to clinical translation remains to be determined. The major challenges, enabling technologies and future roadmap toward clinical translation are summarized in Figure 5, highlighting research priorities that would need to be addressed before safe and effective clinical optogenetic interventions could be considered for neurological and neurodegenerative disorders.

**Figure 5:**
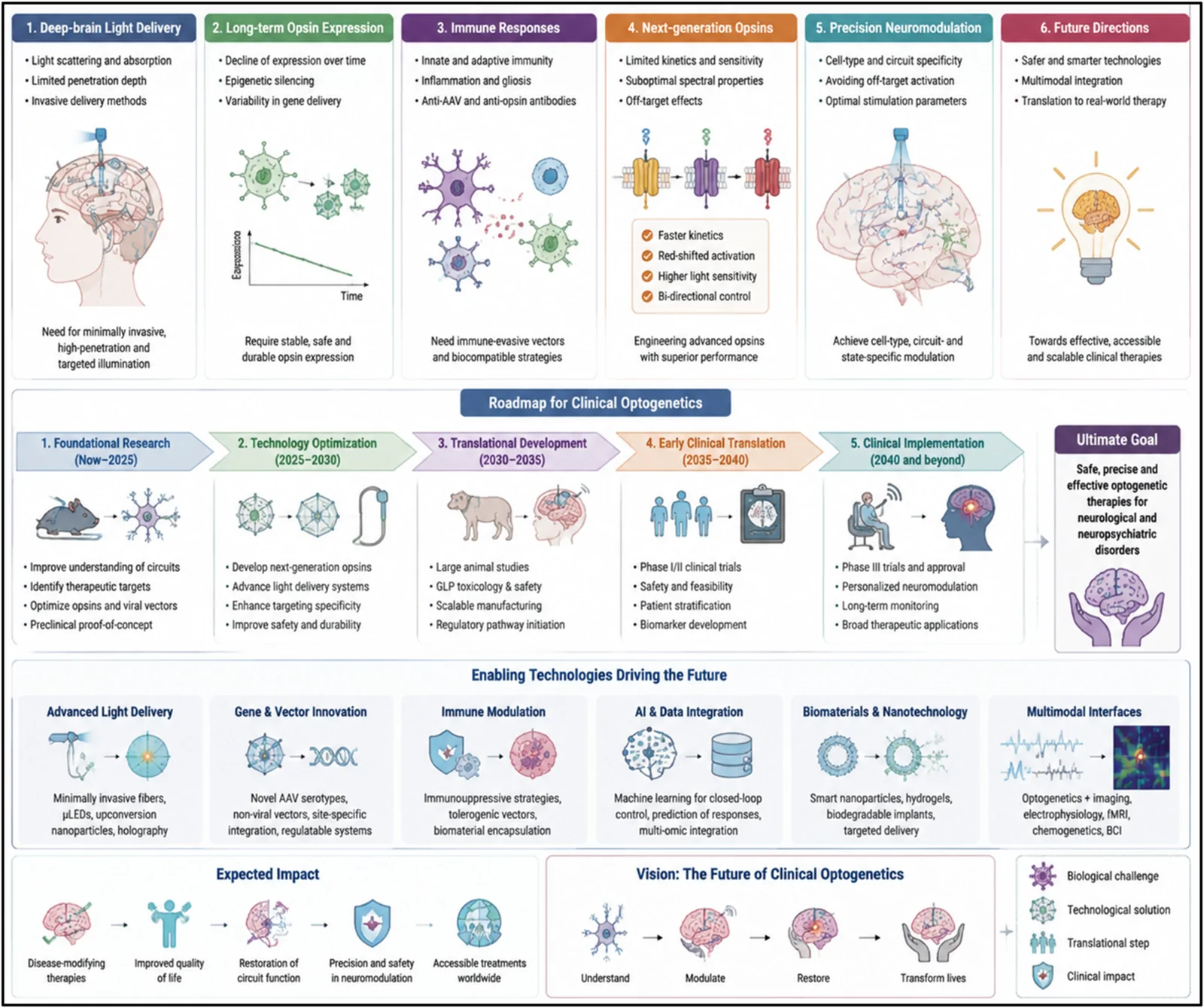
Future roadmap for clinical optogenetics. Current challenges include limited deep-brain light delivery, long-term opsin expression, immune responses, and the need for next generation opsins and precision neuromodulation. Emerging solutions encompass advanced optical technologies, improved viral and nonviral gene delivery systems, biomaterials, nanotechnology, artificial intelligence-assisted adaptive control, and multimodal neurointerfaces. The roadmap outlines the progression from fundamental research and technology optimization to preclinical validation, clinical translation, and personalized neuromodulation, ultimately advancing safe and effective optogenetic therapies for neurological and neurodegenerative disorders.

### 8.1 Deep-brain light delivery

Efficient light delivery to deep-brain regions remains one of the principal challenges in optogenetics. Visible light undergoes significant absorption and scattering within neural tissue, limiting effective stimulation depth and often necessitating invasive implantation of optical fibers. Although fiber-optic systems provide precise spatial targeting, chronic implantation may induce tissue damage, gliosis, inflammation and mechanical instability. Emerging technologies aim to overcome these limitations through minimally invasive approaches. Wireless micro-light-emitting diodes (micro-LEDs), flexible optoelectronic implants, UCNPs and NIR-responsive nanomaterials being investigated to improve access to deeper tissue, although their invasiveness and safety depends on the specific platform (116, 117). In addition, advanced waveguides, holographic light shaping and multiphoton stimulation improve spatial precision and permit simultaneous modulation of multiple neuronal populations. Further development may improve the technical feasibility of deep-brain optogenetic interventions, but long-term safety and clinical utility require dedicated evaluation (16, 109, 116, 117).

### 8.2 Long-term opsin expression

Stable and controlled expression of microbial opsins is essential for chronic neuromodulation. Most current optogenetic systems rely on adeno-associated viral (AAV) or lentiviral vectors to deliver opsin genes; however, long-term expression may vary because of promoter activity, vector tropism, epigenetic regulation and transgene silencing (113, 114). Excessive opsin expression can alter membrane properties, affect neuronal physiology and increase the risk of cytotoxicity. Future strategies focus on improving vector engineering, developing cell type-specific promoters and incorporating inducible gene-expression systems that permit reversible regulation of opsin levels. Advances in genome editing, particularly CRISPR/Cas-based targeted gene insertion, may further improve expression stability while reducing insertional risks. Whether these approaches improve long-term expression control, efficacy or safety in clinically relevant settings remains to be established (15, 112–114).

### 8.3 Immune responses

Immune reactions against viral vectors, microbial opsins, or implanted optical devices remain important barriers to clinical translation. Viral gene delivery can activate innate and adaptive immune pathways, leading to neuroinflammation, reduced transgene expression, and decreased therapeutic efficacy. Likewise, chronic implantation of optical fibers or electronic devices may provoke foreign-body responses characterized by microglial activation, astrocytic gliosis, and fibrotic encapsulation. Current research seeks to improve the biocompatibility of viral vectors and implantable materials while minimizing immunogenicity. Engineered AAV capsids, biodegradable polymers, flexible bioelectronic devices, and transient immunosuppressive strategies have shown promise in reducing inflammatory responses. A better understanding of neuron–glia interactions and host immune mechanisms will be critical for ensuring the long-term safety of optogenetic interventions in humans (16, 118).

### 8.4 Next-generation opsins

The development of next-generation opsins is expanding the versatility and therapeutic potential of optogenetics. First-generation opsins often require high-intensity blue light and exhibit relatively slow kinetics or limited ion selectivity. Protein engineering has produced novel opsins with improved light sensitivity, faster activation and deactivation kinetics, enhanced ion conductance and shifted excitation spectra. Red-shifted opsins, including ChrimsonR and related variants, enable deeper tissue stimulation because red light penetrates biological tissues more effectively than blue light (119, 120). Anion-conducting channelrhodopsins and improved inhibitory pumps provide more efficient neuronal silencing, while bistable opsins permit prolonged modulation using brief light pulses (121). Continued molecular optimization may support multiplexed control of neuronal populations and improve compatibility with emerging light-delivery systems; reduce phototoxicity and safety must be verified for each opsin and stimulation protocol (16, 21).

### 8.5 Precision neuromodulation

The proposed direction for optogenetic research is the integration of genetic targeting, real-time neural recording, computational modeling and adaptive stimulation. Unlike conventional electrical stimulation, optogenetics enables selective modulation of genetically defined neuronal populations while preserving neighboring circuits. Integration with closed-loop control systems, AI-based neural decoding, calcium imaging, electrophysiology and multimodal neuroimaging may permit activity-dependent adjustment of stimulation parameters in appropriately validated experimental platforms. Whether such adaptive approaches improve therapeutic efficacy or reduce adverse effects remain uncertain. Combining optogenetics with pharmacological interventions, electrical stimulation, rehabilitation or regenerative medicine also warrants investigation, although synergistic clinical benefits have not been established. These approaches provide a research framework for evaluating individualized circuit-based interventions rather than evidence of an imminent transition to precision medicine (98).

### 8.6 Future directions

Future research may benefit from combining neuroscience, molecular biology, bioengineering, nanotechnology, AI and regenerative medicine. Improved gene-delivery vectors, wireless optoelectronic devices, NIR-responsive nanomaterials and opsin engineering could address selected technical constraints, although no individual advance is likely to resolve all translational barriers. Stem cell transplantation, CRISPR-mediated gene editing and brain-computer interfaces may also support experimental investigation of circuit repair, but comprehensive functional restoration remains unproven. Clinical translation will require rigorous evaluation of long-term safety, durable gene expression, immune compatibility and ethical considerations associated with genetic manipulation. Standardization of stimulation protocols, optimization of patient selection and well-designed clinical trials will be essential for establishing therapeutic efficacy across neurological disorders. At present, optogenetics remains primarily an experimental platform for causal circuit investigation, with selected applications advancing toward translational evaluation. Its eventual role in clinical diagnosis, neuromodulation, or regenerative therapy will depend on reproducible evidence of patient benefit, acceptable risk, and regulatory feasibility, rather than technological capability alone.

## 9 Conclusion and future perspectives

Optogenetics has transformed neuroscience by enabling precise, reversible and cell-specific manipulation of neuronal activity with exceptional temporal and spatial resolution. By integrating microbial opsins, genetic engineering, optical technologies and systems neuroscience, it has shifted the study of brain function from descriptive observation to causal analysis of neural circuits. Throughout this review, we have highlighted how optogenetics has advanced understanding of neural circuit organization, synaptic plasticity, learning and memory, neurodevelopment, sensory processing, sleep regulation and the molecular mechanisms underlying neurodegenerative disorders. Importantly, optogenetic studies have demonstrated that circuit dysfunction often precedes irreversible neuronal loss, redefining neurodegenerative diseases as disorders of disrupted neural network activity rather than solely progressive neuronal degeneration. Precise modulation of disease relevant circuits has provided critical insights into protein aggregation, synaptic dysfunction, neuroinflammation, mitochondrial impairment, oxidative stress and regulated cell death, while demonstrating functional improvements in preclinical models of AD, PD, HD, ALS, MS and stroke. However, these findings primarily represent proof-of-concept evidence for circuit modulation rather than confirmed therapeutic efficacy in human patients. Thus, the current role of optogenetics remains predominantly focused on elucidating disease mechanisms, identifying therapeutic targets, and evaluating circuit-based interventions rather than serving as an established clinical treatment strategy. Despite remarkable progress, several challenges continue to limit clinical translation, including safe and efficient gene delivery, long-term opsin expression, immune responses, limited light penetration into deep-brain regions, device biocompatibility and regulatory considerations. Advances in red-shifted and near-infrared responsive opsins, wireless photonic implants, minimally invasive optical devices, nonviral gene delivery systems and nanotechnology may facilitate improved spatial targeting, reduced invasiveness and enhanced therapeutic feasibility; however, their clinical effectiveness requires further validation through long-term safety studies and human investigations. Furthermore, integration of optogenetics with single cell and spatial omics, genetically encoded biosensors, artificial intelligence, closed-loop neuromodulation, brain-computer interfaces and gene editing technologies may offer promising opportunities for comprehensive characterization and modulation of disease specific neural circuits, although most of these approaches remain at experimental or preclinical stages. In conclusion, optogenetics has evolved from an experimental neuroscience tool into a powerful platform for precision circuit analysis and an emerging, yet still investigational, approach for future neuromodulation strategies. Continued technological innovation and multidisciplinary validation may support future clinical assessment; however, practical implementation will depend on overcoming biological, engineering and regulatory barriers and demonstrating meaningful patient outcomes. Accordingly, potential diagnostic or therapeutic applications in neurodegenerative disorders should be regarded as research objectives rather than anticipated clinical achievements.
